# An overview of the BIOASQ large-scale biomedical semantic indexing and question answering competition

**DOI:** 10.1186/s12859-015-0564-6

**Published:** 2015-04-30

**Authors:** George Tsatsaronis, Georgios Balikas, Prodromos Malakasiotis, Ioannis Partalas, Matthias Zschunke, Michael R Alvers, Dirk Weissenborn, Anastasia Krithara, Sergios Petridis, Dimitris Polychronopoulos, Yannis Almirantis, John Pavlopoulos, Nicolas Baskiotis, Patrick Gallinari, Thierry Artiéres, Axel-Cyrille Ngonga Ngomo, Norman Heino, Eric Gaussier, Liliana Barrio-Alvers, Michael Schroeder, Ion Androutsopoulos, Georgios Paliouras

**Affiliations:** 1 Biotechnology Center, TU Dresden, 01307 Tatzberg 47-49, Dresden, Germany; 2 Transinsight GmbH, Tatzberg 47-49, Dresden, 01307 Germany; 3 NCSR Demokritos, Ag. Paraskevi, Athens, 60228 Greece; 4 Athens University of Economics and Business, Patission 76, Athens, 10434 Greece; 5 Université Pierre et Marie Curie-Paris 6, 4 Place Jussieu, Paris, 75005 France; 6 Universität Leipzig, Augustusplatz 10, 04109 Leipzig, Germany; 7 Université Joseph Fourier, 621 Avenue Centrale, Saint-Martin-d’Héres, 38041 France

**Keywords:** BioASQ Competition, Hierarchical Text Classification, Semantic indexing, Information retrieval, Passage retrieval, Question answering, Multi-document text summarization

## Abstract

**Background:**

This article provides an overview of the first BioASQ challenge, a competition on large-scale biomedical semantic indexing and question answering (QA), which took place between March and September 2013. BioASQ assesses the ability of systems to semantically index very large numbers of biomedical scientific articles, and to return concise and user-understandable answers to given natural language questions by combining information from biomedical articles and ontologies.

**Results:**

The 2013 BioASQ competition comprised two tasks, Task 1a and Task 1b. In Task 1a participants were asked to automatically annotate new PubMed documents with MeSH headings. Twelve teams participated in Task 1a, with a total of 46 system runs submitted, and one of the teams performing consistently better than the MTI indexer used by NLM to suggest MeSH headings to curators. Task 1b used benchmark datasets containing 29 development and 282 test English questions, along with gold standard (reference) answers, prepared by a team of biomedical experts from around Europe and participants had to automatically produce answers. Three teams participated in Task 1b, with 11 system runs. The BioASQ infrastructure, including benchmark datasets, evaluation mechanisms, and the results of the participants and baseline methods, is publicly available.

**Conclusions:**

A publicly available evaluation infrastructure for biomedical semantic indexing and QA has been developed, which includes benchmark datasets, and can be used to evaluate systems that: assign MeSH headings to published articles or to English questions; retrieve relevant RDF triples from ontologies, relevant articles and snippets from PubMed Central; produce “exact” and paragraph-sized “ideal” answers (summaries). The results of the systems that participated in the 2013 BioASQ competition are promising. In Task 1a one of the systems performed consistently better from the NLM’s MTI indexer. In Task 1b the systems received high scores in the manual evaluation of the “ideal” answers; hence, they produced high quality summaries as answers. Overall, BioASQ helped obtain a unified view of how techniques from text classification, semantic indexing, document and passage retrieval, question answering, and text summarization can be combined to allow biomedical experts to obtain concise, user-understandable answers to questions reflecting their real information needs.

**Electronic supplementary material:**

The online version of this article (doi:10.1186/s12859-015-0564-6) contains supplementary material, which is available to authorized users.

## Background

BIOASQ is an EU-funded support action [1] to set up a challenge on biomedical semantic indexing and question answering (QA). Participants are required to semantically index documents from large-scale biomedical repositories (e.g., MEDLINE) and to assemble information from multiple heterogeneous sources (e.g., scientific articles, ontologies) in order to compose answers to real-life biomedical English questions. BIOASQ addresses a central problem biomedical knowledge workers face: to synthesise and filter information from multiple, large, fast-growing sources. Existing search engines (e.g., PUBMED, GOPUBMED [2,3]) only partially address this need. They focus on a limited range of resources (e.g., only MEDLINE articles and concepts from GENE ONTOLOGY or MESH), whereas multiple sources (e.g., including specialised drug databases and ontologies) often need to be combined. Furthermore, they mostly retrieve possibly relevant texts or structured information, which the users then have to study, filter, and combine by themselves to obtain the answers they seek. By contrast, QA systems aim to directly produce answers [4]. Semantic indexing, i.e., annotating documents with concepts from established semantic taxonomies or, more generally, ontologies, provides a means to combine multiple sources and facilitates matching questions to answers. In recent years, many methods have been developed that utilize existing ontology structures and concepts to index documents and perform semantic search [5]. Current semantic indexing, however, in the biomedical domain is largely performed manually, and needs to be automated to cope with the vast amount of new information that becomes available daily. At the same time, current semantic indexing and QA methods require more research to reach a level of effectiveness and efficiency acceptable by biomedical experts. BIOASQ sets up ambitious, yet feasible and clearly defined challenge tasks, intended to lead to integrated, efficient, and effective semantic indexing and QA methods for the biomedical domain. In addition, BIOASQ helps in the direction of establishing an evaluation framework for biomedical semantic indexing and QA systems. It does so by developing realistic, high-quality benchmark datasets and adopting (or refining) existing evaluation measures for its challenge tasks.

**Figure 1:**
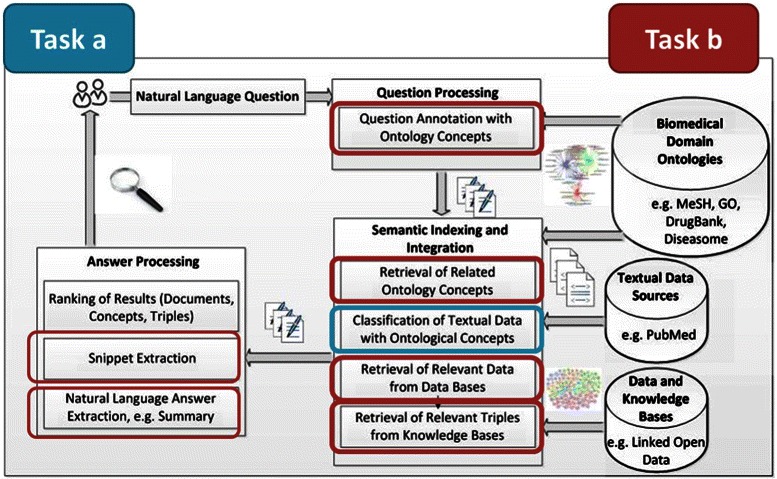
Overview of semantic indexing and question answering in the biomedical domain. The BIOASQ challenge focuses in pushing systems towards implementing pipelines that can realize the workflow shown in the figure. Starting with a variety of data sources (lower right corner of the figure), semantic indexing and integration brings the data into a form that can be used to respond effectively to domain specific questions. A semantic QA system associates ontology concepts with each question and uses the semantic index of the data to retrieve the relevant pieces of information. The retrieved information is then turned into a concise user-understandable form, which may be, for example, a ranked list of candidate answers (e.g., in factoid questions, like *“What are the physiological manifestations of disorder Y?”*) or a collection of text snippets, ideally forming a coherent summary (e.g., in *“What is known about the metabolism of drug Z?”*). The figure also illustrates how these steps are mapped to the BIOASQ challenge tasks. With blue, Task 1a is depicted, while red depicts Task 1b.

Figure 1 provides a general overview of biomedical semantic indexing and QA in BIOASQ. Other recent approaches also follow a similar approach [4,6]. Starting with a variety of data sources (lower right corner of the figure), semantic indexing and integration brings the data into a form that can be used to respond effectively to domain-specific questions. A semantic QA system associates ontology concepts with each question and uses the semantic index to retrieve relevant texts (documents or abstracts, e.g., from PUBMED or PUBMED CENTRAL) to retrieve pieces of structured information (e.g., Linked Open Data triples) and relevant documents (or abstracts, e.g., from PUBMED). This is depicted in the middle of the figure, by the processes included in the *Question Processing* and *Semantic Indexing and Integration* boxes. The retrieved information is then turned into a concise, user-understandable form, which may be, for example, a ranked list of candidate answers (e.g., in factoid questions, like *“What are the physiological manifestations of disorder Y?”*) or a collection of text snippets (ideally forming a coherent summary) jointly providing the requested information (e.g., in *“What is known about the metabolism of drug Z?”*). More precisely, the BIOASQ challenge evaluates the ability of systems to perform: (1) large-scale classification of biomedical documents onto ontology concepts, in order to automate semantic indexing, (2) classification of biomedical questions on the same concepts, (3) integration of relevant document snippets, database records, and information (possibly inferred) from knowledge bases, and, (4) delivery of the retrieved information in a concise and user-understandable form.

To realize the challenge, BIOASQ organized two tasks, namely Task 1a (covering point number 1 from the aforementioned list) and Task 1b (covering the rest of the points from the aforementioned list). In Task 1a, named “*Large-scale online biomedical semantic indexing*”, participants were asked to classify new abstracts written in English, as they became available online, before MEDLINE curators annotated (in effect, classified) them manually; at any point in time there was usually a backlog of approximately 10,000 non-annotated abstracts. The classes came from the MESH hierarchy, i.e., the subject headings that are currently used to manually index the abstracts. As new manual annotations became available, they were used to evaluate the classification performance of participating systems (that classified articles before they were manually annotated), using standard information retrieval (IR) measures (e.g., precision, recall, accuracy), as well as hierarchical variants of these measures. In Task 1b, named “*Introductory biomedical semantic QA*”, participants were asked to annotate input natural language questions with biomedical concepts, and retrieve relevant documents, snippets and triples (Phase A). Finally, participants were asked to find and report the answers to the questions (Phase B), given as additional input the golden responses of the Phase A. The answers of the systems were compared against model answers in English constructed by biomedical experts, using evaluation measures from QA and summarization. A running example of a participating system that answers a natural language question, progressing through the BIOASQ competition tasks, in order to illustrate how the various steps of the competition are combined to allow systems to address natural language QA is provided in Additional file 1. The benchmark datasets that contain the development and evaluation questions, as well as the gold standard (reference) answers, are made publicly available. The gold standard answers were produced by a team of biomedical experts from research teams around Europe. Established methodologies from QA, summarization, and classification were followed to produce the benchmarks and evaluate the participating systems.

### Related NLP and QA evaluations

Since the late 1990s, QA research has benefited significantly from competitions organised in the context of large conferences, such as the Text Retrieval Conference (TREC) [7]. TREC’s QA track [8] initially focused mostly on factoid questions, while TREC’s Genomics track focused on retrieval tasks for genomics data, including gene sequences and supporting documentation, such as research papers and lab reports [9]. More recent research, however, has also explored questions that ideally require a summary of the most important information in a cluster of relevant documents, gradually bringing QA for document collections closer to text summarization. This trend is also evident in the more recent Text Analysis Conference (TAC) series, which has included challenge tasks such as query-focused (or topic-based) summarization [10]. QA systems for document collections typically search for answers in clusters of documents returned by information retrieval (or Web search) engines. Hence, information retrieval (IR) tasks are also relevant to QA.

Non-English QA competitions have also been organised in the past; for example, in the context of *NTCIR* [11]. Also, cross-lingual QA and document retrieval competitions have been held in *CLEF* [12,13]. Apart from QA and IR challenges for document collections, “semantic search” challenges are also beginning to appear: ***Semantic search challenge:*** Finding answers to keyword queries in Linked Data (in the form of RDF triples). The data originate from the 2009 Billion Triple Challenge. (Ran in 2011.)

As already noted, the semantic indexing task of BIOASQ asks for documents and questions to be annotated with concepts from biomedical hierarchies. The following hierarchical classification challenges are, hence, also relevant: ***LSHTC:*** The Large Scale Hierarchical Text Classification Challenges [14], which were organised by members of the BIOASQ consortium, provided benchmarks (based on *Wikipedia* and the *DMOZ* Open Directory Project), as well as a common evaluation framework for hierarchical classifiers [15]. (Ran from 2010 to 2014.) ***Joint Rough Sets (JRS) symposium’s special event:*** Topical classification of biomedical articles, based on MESH concepts automatically assigned to articles by a tagger developed by the organisers [16]. (Ran in 2012.)

Finally, the Special Interest Group on Biomedical Natural Language Processing (SIGBIOMED) of the Association for Computational Linguistics (ACL) organises the *BioNLP* annual workshops, which focus mostly on information extraction from biomedical documents (e.g., recognising named entities, particular relations between named entities, or biological events mentioned in scientific articles). The 2009 and 2011 *BioNLP* workshops featured shared tasks in these areas [17]. Similar biomedical information extraction and data mining challenges have been (and continue to be) organised in the context of *BioCreative*, with a recent additional emphasis on helping curators of biomedical document collections (e.g., prioritising articles to be manually curated) [18].

Overall, although there have been several competitions relevant to BIOASQ, which required searching large-scale document collections or structured datasets, question answering or text summarization, and hierarchical classification or semantic indexing, very few of them were directly concerned with biomedical data. Furthermore, none of them required the participants to answer biomedical natural language questions by searching in both structured data (e.g., databases, ontologies, and the Linked Open Data cloud) and unstructured data (e.g., biomedical articles), and none of them pushed at the same time towards matching questions to answers at the conceptual level, i.e., by using concepts from domain ontologies to annotate both questions and answers. Hence, BIOASQ has a broader scope, incorporating hierarchical classification, text and passage retrieval, retrieving RDF triples, QA for exact answers, multi-document summarization, and natural language generation.

## Description of the BIOASQ Tasks

### Description of Task 1a

Task 1a, titled *“Large scale online biomedical semantic indexing”*, deals with large scale classification of biomedical documents into ontology concepts. The purpose is to investigate the performance of the state of the art methods and the new methodologies proposed by participants compared to the manual annotation that is widely used in public databases.

As new articles are uploaded on a daily basis in MEDLINE and are annotated manually with concepts from the MESH hierarchy, MEDLINE offers the means to assess the performance in a real large scale setting. Task 1a takes advantage of two observations of the PUBMED workflow: a large number of uploaded articles (approximately 4,000 on a daily basis), and from them a sufficient fraction are annotated in a relatively short time suitable for the BIOASQ challenge (approximately 10*%* within two weeks, and more than 50*%* within 12 weeks). Similar observations have also been reported in the past [19,20]. On this basis, the time frame between the first appearance of an article in PUBMED and its indexing with MESH terms is used by the BIOASQ team in order to prepare test sets that consist of non-annotated articles. Many recently published approaches are applicable to this BIOASQ task, e.g., [20-22].

During the challenge period, test sets are released regularly, i.e., once per week. This allows participants to improve their systems by taking into account the partial evaluation results that become available, and allows the participants to enter the challenge at any stage. The articles that are selected for each test set are filtered based on the journal average annotation time to ensure a short annotation period. Participants have 21 hours from the release of the test set to submit their system’s estimations of MESH terms for the released articles. The evaluation of the participating systems is performed incrementally each time new annotations become available from MEDLINE by human curators. As training data, the participants are given all of the previously annotated PUBMED articles with their respective MESH annotations, so that the participating teams could tune their annotation methods to the specific task.

#### Creation of benchmark datasets for Task 1a

In Task 1a the data that are available to the participants consist of biomedical articles published in MEDLINE. Specifically, for each article in the training data, BIOASQ provides its title and abstract as it appears in MEDLINE and the MESH labels assigned to it. In the testing phase of the challenge the data contain only the title, the abstract, the journal and the year of the corresponding article without any further information. The articles are provided in their raw format (plain text) as well as in a pre-processed one (bag of words with weights). The subsection ‘An extract from the training data of Task1a’ presents an example of two articles extracted from the BIOASQ benchmark training data. The format used for the distribution of the data is the *JavaScript Object Notation* [23] (*JSON*).

### An extract from the training data of Task1a



For the creation of the training data, all of the MEDLINE articles that were assigned MESH labels were collected and distributed to the authors. There were only two requirements regarding the collection of the articles: (1) that the articles had a title, an abstract and MESH labels assigned, and, (2) that their indexing with MEDLINE was made before the beginning of the Task 1a, i.e., in our case this date was set to March 1st, 2013. Table 1 presents the statistics of the training data set for Task 1a.

**Table 1:** **Basic statistics about the training data for Task1a**

**Number of articles**	10,876,004
**Number of unique ** MESH ** labels**	26,563
**Average number of ** MESH ** labels per article**	12.55
**Size in GB**	22

For the creation of the test data three batches were designed, each containing 6 test datasets. Each week a test dataset was distributed to the participants. Thus, each batch was 6 weeks long. The following requirements were used for the creation of the test datasets: (1) that the articles should have a title, an abstract, but no MESH headings assigned to them yet, (2) that the articles should have MESH headings assigned in few weeks time after their release, (3) the articles that have been released in a previous dataset should not be released in any other future test set. To fulfil all three requirements, a web service was set up to automatically fetch from MEDLINE the articles of this kind. Requirements (1) and (3) are easy to satisfy with a PUBMED query. For requirement (2) we analyzed the average time it takes to assign MESH labels to articles, per journal, for all of the MEDLINE indexed journals. From this analysis, only the journals for which the average assignment of MESH labels took up to 90 days were kept. In total, 1,993 such journals were used. This list of selected journals can be found in Additional file 2. Hence, requirement (2) was now reduced to the selection of articles that are published in one of the journals in the list. An example of a PUBMED query that is used by the BIOASQ web service to create test datasets can be found in Additional file 3. In collaboration with NLM we used a set of additional filters that excluded articles that are editorials, comments, reviews, letters, and news from the resulting article list.

With the satisfaction of all three requirements, the test datasets would comprise unique articles for which the MESH terms were not known at the time of the release, but would become known on average up to 90 days after their release date, thus, allowing the BIOASQ consortium to evaluate Task 1a on time before the end of the challenge. In Table 2 we present the number of articles of each test dataset in each batch of the evaluation procedure. The numbers in parentheses are those articles of the corresponding test dataset that were annotated with MESH labels by the NLM curators by the time that the Task 1a results were frozen (September 2013).

**Table 2:** **Number of articles for each test dataset in each batch**

**Week**	**Batch 1**	**Batch 2**	**Batch 3**
1	1,942 (1,553)	4,869 (3,414)	7,578 (2,616)
2	830 (726)	5,551 (3,802)	10,139 (3,918)
3	790 (761)	7,144 (3,983)	8,722 (2,969)
4	2,233 (586)	4,623 (2,360)	1,976 (1,318)
5	6,562 (5,165)	8,233 (3,310)	1,744 (1,209)
6	4,414 (3,530)	8,381 (3,156)	1,357 (696)
Total	16,763 (12,321)	38,801 (20,025)	31,570 (12,726)

In parentheses the articles that have been annotated by the curators by the time of the Task 1a evaluation (September 2013).

#### Evaluation measures for Task 1a

**Figure 2:**
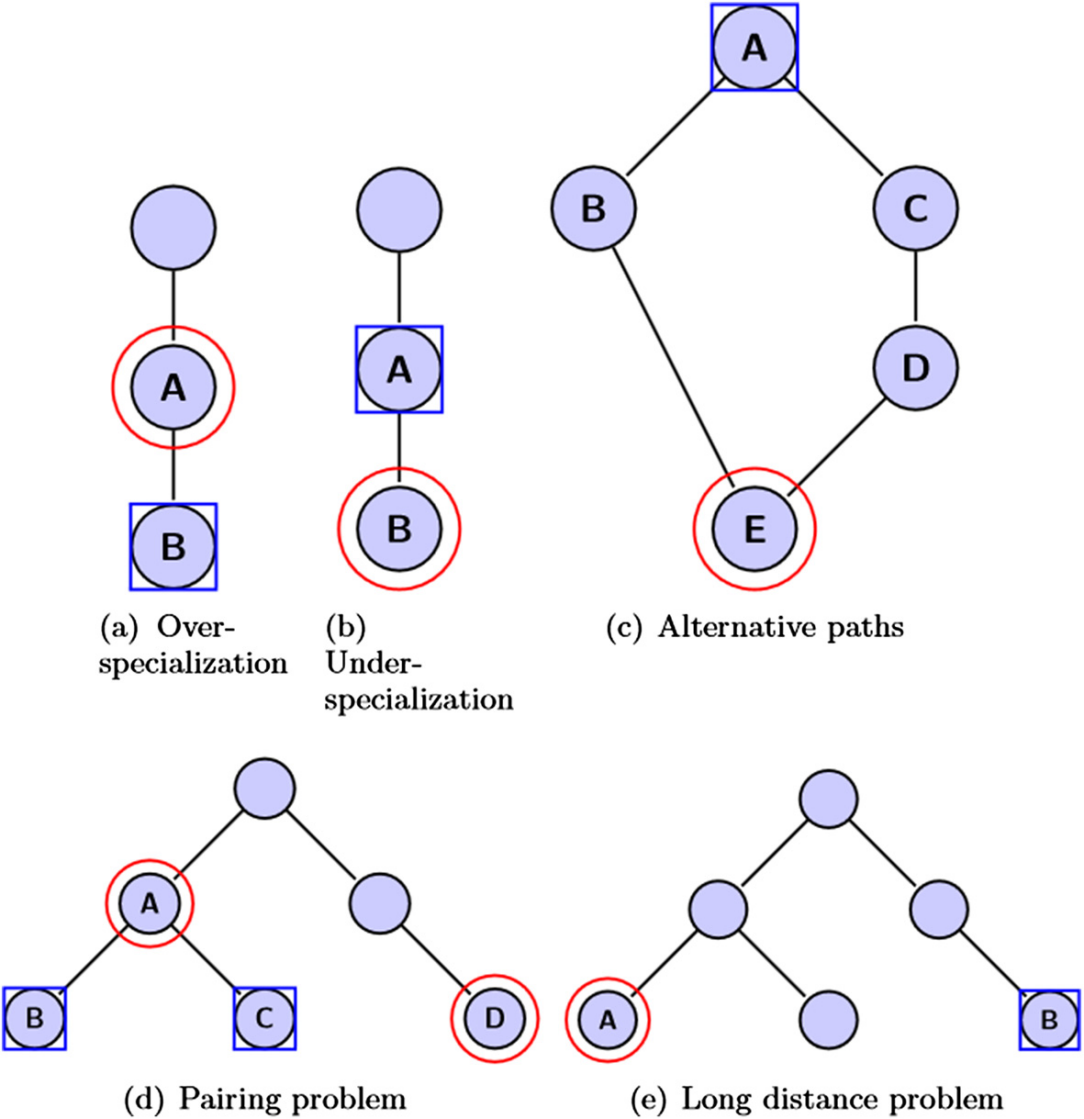
Interesting cases when evaluating hierarchical classifiers: **(a)** over-specialization, **(b)** under-specialization, **(c)** alternative problems, **(d)** pairing problem, **(e)** long distance problem. Nodes surrounded by circles are the true classes while the nodes surrounded by rectangles are the predicted classes. LCaF ia based on the notion of adding all ancestors of the predicted (rectangles) and true (circles) classes. However, adding all the ancestors has the undesirable effect of over-penalizing errors that happen to nodes with many ancestors. Thus, LCaF uses the notion of the Lowest Common Ancestor to limit the addition of ancestors.

For the evaluation of the participating systems in Task 1a, two measures, a flat and a hierarchical, are considered. The main difference between them is that the latter takes into account the relations in the given hierarchy, penalizing more heavily misclassifications in distant branches of the hierarchy. Both measures are applicable for the evaluation of all types of classifier. The flat measure that is used is the micro-F1 measure, which is a label-based measure [24]. The hierarchical measure is the *LCaF* [25]. In Figure 2 we illustrate the basic concept of the measure. All the details of the evaluation measures for Task 1a can be found in Appendix A.

#### Baseline systems for Task 1a

For the purposes of BIOASQ Task 1a, three baselines were utilized, namely the BIOASQ_BASELINE, the MTI, and its special design, the MTIFL. The first baseline, the BIOASQ_BASELINE, is the *Attribute Alignment Annotator* developed by Doms [26]. It is an unsupervised method, based on the Smith-Waterman sequence alignment algorithm [27] and can recognize terms from MESH and GENE ONTOLOGY in a given text passage. The annotator first pre-processes both the ontology terms and the text by tokenizing them, removing the stop words and stemming the remaining terms^a^. Then the term stems are mapped onto the text stems using the local sequence alignment algorithms [27]. Insertions, deletions and gaps are penalized. The information value of terms calculated over the whole ontology is also taken into account during the alignment process of ontology terms with text terms, in a similar manner as the inverse document frequency score is used for the *TF-IDF* weighting of terms.

The second baseline for Task 1a is the NLM’s *Medical Text Indexer* [28] (MTI). MTI has been providing indexing recommendations for PUBMED based on the MESH vocabulary since 2002. MTI produces both semi- and fully-automated indexing recommendations based on MESH. MEDLINE indexers and revisers consult MTI recommendations for approximately 58*%* of the articles they index. MTI provides an ordered list of MESH main headings, subheadings and check tags. For the purposes of BIOASQ Task 1a, only the suggested MESH main headings are used. Its main components are: (1) the *MetaMap Indexing*, which applies a ranking function to concepts found by *MetaMap* in the input text, (2) the identification of PUBMED related citations, which finds the closest documents to the input text, (3) the identification of the closest MESH headings to the identified UMLS concepts, (4) the extraction of MESH descriptors, (5) the clustering and ranking of the extracted MESH headings, and, (6) the post-processing, which is mostly related to the inclusion of check tags.

In 2011 MTI’s role was expanded by designating it as the first-line indexer (MTIFL) for a few journals; today the MTIFL workflow includes about 100 journals and continues to increase. For MTIFL journals, MTI indexing is treated like human indexing, i.e., being used instead of human indexing. The MTIFL comprises the third BIOASQ Task 1a baseline, and its main difference to the MTI indexer is the level of filtering it applies for the final recommendation of MESH headings. MTIFL is based on a *Balanced Recall/Precision Filtering*, which looks at the compatibility and context of the recommendation based on what path(s) made the recommendation and provides a good balance between number of recommendations and the filtering out of good recommendations, while MTI utilizes the *High Recall Filtering*, and tends to provide a list of approximately 25 recommendations with most of the good recommendations near the top of the list.

### Description of Task 1b

Task 1b, titled *“Biomedical Semantic Question Answering”*, examines systems’ ability to annotate questions with concepts from relevant ontologies and depending on the type of the question, return “exact” or paragraph-sized “ideal” answers. The benchmark dataset comprises the questions, their answers, and the related documents, concepts, triples, and statements from designated repositories. It was created by the BIOASQ biomedical expert team, using the BIOASQ annotation tool that will be described later.

The task was organised in two phases; (a) *Phase A*: The BIOASQ team released questions from the benchmark datasets. The participating systems had to respond with relevant concepts from designated terminologies and ontologies, relevant articles in English from designated article repositories, relevant snippets from the relevant articles, and relevant *RDF* triples (statements) from designated ontologies. The participating systems were allowed to return at most 100 concepts, 100 documents, 100 snippets and 1,000 *RDF* triples per question; (b) *Phase B*: The BIOASQ team released questions and gold (correct) relevant concepts, articles, snippets, and *RDF* triples (statements) from the benchmark datasets. The participating systems had to respond with “exact” answers, i.e., *“yes”* or *“no”* in the case of *yes/no questions*, named entities in the case of *factoid questions*, list of named entities in the case of *list questions* and nothing in the case of summary questions, and “ideal” answers (i.e., paragraph-sized summaries) for all types of questions. We call them “ideal” because it is what a human would expect as an answer by a peer biomedical scientist. For the synthesis of the answers, the systems were allowed to use the provided gold documents, concepts, snippets and statements which were found related by the experts. Systems were also allowed to use the annotations their systems suggested in *Phase A*, or apply their *Phase A* systems to produce annotations from any additional resources.

#### Creation of benchmark datasets for Task 1b

In Task 1b, the benchmark datasets contain development and test questions, in English, along with golden standard (reference) answers. The benchmark datasets have been constructed by the BIOASQ team of biomedical experts. As in Task 1a, the datasets follow the *JSON* format. More specifically, each dataset (development and test sets) contains an array of questions where each question (represented as an object in the *JSON* format) is constructed as shown in ‘The format of the training data of Task1b’ subsection.

### The format of the training data of Task1b



The distributed questions can be of four types: (1) *“Yes/No”*, (2) *factoid*, (3) *list*, and, (4) *“summary”*. For each of the question types, there can be both “ideal” and “exact” answers expected, besides the *“summary”* questions, where only “ideal” answers are expected. Examples of the four question types, along with description of what is expected and the golden answers that are provided by the experts are presented in Table 3. In all cases, the ideal answers are restricted to a length of 200 words. A length restriction also applies in the case of the “exact” answers, where each of the returned entities within the answer can be up to 100 characters long.

**Table 3:** **Types of questions in Task 1b and respective examples along with the golden answers in each case**

**Question**	**Required**	**Example**	**Golden exact**	**Golden Ideal**
**type**	**answer**	**question**	**answer**	**answer**
Yes/No	Exact + Ideal	Is miR-21 related to carcinogenesis?	Yes	Yes. It has been demonstrated in several experimental studies that miR-21 has oncogenic potential, and is significantly disregulated in numerous types of cancer. Therefore, miR-21 is closely related to carcinogenesis.
Factoid	Exact + Ideal	Which is the most common disease attributed to malfunction or absence of primary cilia?	“autosomal recessive polycystic kidney disease”	When ciliary function is perturbed, photoreceptors may die, kidney tubules develop cysts, limb digits multiply and brains form improperly. Malformation of primary cilia in the collecting ducts of kidney tubules is accompanied by development of autosomal recessive polycystic kidney disease.
List	Exact + Ideal	Which human genes are more commonly related to craniosynostosis?	[“MSX2”, “RECQL4”, “SOX6”, “FGFR1”, “FGFR2”, “FGFR”]	The genes that are most commonly linked to craniosynostoses are the members of the Fibroblast Growth Factor Receptor family FGFR3 and to a lesser extent FGFR1 and FGFR2. Some variants of the disease have been associated with the triplication of the MSX2 gene and mutations in NELL-1.
Summary	Ideal	What is the mechanism of action of abiraterone?	-	Abiraterone acts by inhibiting cytochrome P450 170̆3b1-hydroxylase (CYP17A1), a critical step in androgen biosynthesis, thus leading to inhibition of androgen biosynthesis.

In both phases of Task 1b, the question type is always distributed along with the actual natural language question. The development set of questions contains both the “ideal” and “exact” answers for the 29 development questions, so that the participants can train on how to answer each type of questions. In Table 4 we present the statistics of the released development (training) and test questions, in the framework of BIOASQ Task 1b.

**Table 4:** **Statistics of the training and test data for Task 1b**

	**Training**	**Test set 1**	**Test set 2**	**Test set 3**
	**data**			
**Questions**	29	100	100	82
**Yes/No**	8	25	26	26
**Factoid**	5	18	20	16
**List**	8	31	31	23
**Summary**	8	26	23	17
**Avg #concepts**	4.8	5.3	6.0	12.9
**Avg #documents**	10.3	11.4	12.1	5.4
**Avg #snippets**	14.0	17.1	17.4	15.9
**Avg #triples**	3.6	21.8	5.5	4.5

#### Evaluation measures for Task 1b

##### Evaluation process and measures for Task 1b Phase A

**Figure 3:**
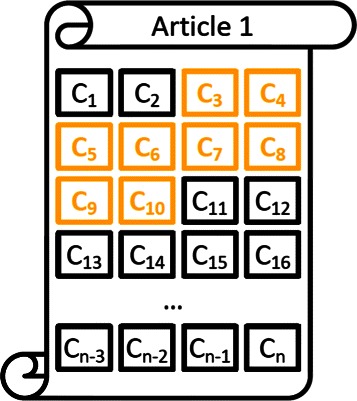
An illustration for the article-offset pairs. An article-offset pair example. Article 1 has *n* characters and a golden snippet starting at offset 3 and ending at offset 10.

In Phase A, the participants were provided with English questions. For each question, each participating system was required to return a list of relevant concepts, a list of relevant articles, a list of relevant text snippets, and a list of relevant RDF triples. For each question, the biomedical experts have produced the gold (correct) sets of concepts, articles, snippets, and triples. Given this setup, for each system, the lists of returned concepts, articles, snippets, and triples of all the questions were evaluated using the *mean average precision* (MAP) measure, which is widely used in information retrieval to evaluate ranked lists of retrieved items. In the case of snippets, however, a special consideration took place for the evaluation, on the basis that a returned snippet may overlap with one or more golden snippets, without being identical to any of them. Therefore, in the case of the snippets, the definition of precision and recall was modified to consider a snippet as a set of article-offset pairs. Figure 3 illustrates what we mean by article-offset pairs. A snippet is determined by the article it comes from and by the offsets (positions) in the article of the first and last characters of the snippet. The details of these modifications for the snippets, as well as of MAP and other additional measures used for the evaluation of the systems in Phase A of Task 1b can be found in Appendix A.

##### Evaluation process and measures for Task 1b Phase B

In Phase B, the participants were provided with the same questions as in Phase A, but this time they were also given the golden (correct) lists of concepts, articles, snippets, and triples of each question. For each question, each participating system returned an “ideal” answer, i.e., a paragraph-sized summary of relevant information. In the case of *“yes/no”*, *factoid*, and *list* questions, the systems also had to return “exact” answers. The evaluation of the “exact” answers was conducted using *accuracy* (ACC) for the *“yes/no”* and *factoid* questions, while for the *list* questions *precision* (*P*), *recall* (*R*), and *F-measure* (*F*_1_) was used. The “ideal” answers of the systems were evaluated both manually (by the BIOASQ team of biomedical experts) and automatically (by comparing them to the golden “ideal” answers). The official scores were based on the manual evaluation; the automatic evaluation was performed mostly to explore how well automatic evaluation measures (e.g., from multi-document text summarization) correlate with the scores of the biomedical experts. For the automatic evaluation the ROUGE score was used [29]. The details of all measures used for the evaluation of the systems in Phase B of Task 1b are explained in detail in Appendix A.

#### Baseline systems for Task 1b

For the creation of a baseline approach in Task 1B Phase A, two simple methods were considered. Given a question, for each of the required type of returned info, i.e., documents, snippets, concepts and triples, the search services that are made available to the participants were used to query the underlying resources, and the top-50 and top-100 results respectively were kept as baseline answers for each type of results. Hence, the first baseline (*Top-50*) returns (at maximum) the top-50 documents, snippets, concepts and triples (e.g., maximum 50 for each type), and the second baseline returns respectively (at maximum) the top-100.

As baseline in Task 1B Phase B, different approaches were created to produce “ideal” and “exact” answers for the different types of questions (i.e., yes/no, factoid, list, summary). Concerning “ideal” answers, two different summarization approaches were used. The first one greedily selects the content of the answer while the second utilizes Integer Linear Programming for the same purpose. They both employ a Support Vector Regression model to evaluate the quality of the content [30]. The summaries produced are then used to provide “exact” answers for yes/no questions; each summary is scanned for ‘positive’ and ‘negative’ words (as listed in a sentiment lexicon), and respond ‘yes’ or ‘no’ if the positive words are more or fewer, respectively, than the negative ones. To produce “exact” answers for list and factoid questions the baseline approach combines a set of scoring schemes that attempt to prioritize the concepts that answer the question by assuming that the type of the answer aligns with the lexical answer type (type coercion). The approach is described in detail in the work of Weissenborn et al. [31].

Finally, as a summary, the roll-out of the challenge can be found in Additional file 4, where the overall process of the challenge organisation and execution is illustrated in a single schema.

## Results and discussion

In this section, the results of the participating systems are presented. Overall, in Task 1a there was high participation and the task was very successful. One of the participating systems in Task 1a performed consistently better than NLM’s MTI system [32]. As a result, NLM has reported recently that the MTI indexer was enhanced with ideas from the winners of Task 1a, and that the BIOASQ challenge has been a tremendous benefit for NLM by expanding their knowledge of other indexing systems [33]. In the case of Task 1b, the participation was low and the performance of the systems was not very high, mostly due to the complexity and the difficulty of the task. However, the manual evaluation of the “ideal” answers of the participating systems received relatively high scores, which is a promising outcome, as it means that the participating systems were able to produce high quality summaries that could answer part, or in some cases the whole, of the given natural language questions.

In the following, systems are referred by the name submitted when registering to the challenge. Contestants participated through one or more systems. Prizes were awarded to the winners of both tasks of the challenge; a total of 12,000 Euros was distributed to the winners, and a special prize was awarded to the team with the overall best contribution^b^. A description of the system’s methodology was not a requirement for participating. Table 5 presents the correspondence of the BIOASQ system names with system’s reference publications, when available. Systems that participated in less than 4 test sets in each batch are not reported^c^.

**Table 5:** **Correspondence of reference and submitted systems for Task1a**

**Reference**	**Systems**
[32]	system1, system2, system3, system4, system5
[37]	cole_hce1, cole_hce2, utai_rebayct, utai_rebayct_2
[45]	mc1, mc2, mc3, mc4, mc5
[48]	Wishart-*
[49]	RMAI, RMAIP, RMAIR, RMAIN, RMAIA
Baselines [28,31]	MTIFL, MTI, bioasq_baseline

### Timeline of the competition for the participants

The training data for Task 1a were officially released on March 18th, 2013. On April 15th a dry-run set was released to familiarize the participants with the downloading of the test sets and the uploading of the results. A week after, the official test sets started to be released. Hence, from April 22nd to August 26th, every week at a specific day and time a test set was released, and the participants had 21 hours to upload the results. In total there were 18 test sets, split into three batches of 6. In the case of Task 1b, the training data were released on June 6th, and on June 26th the first of the three test sets was released for phase A, and the next day, i.e., June 27th, for phase B. The second test set was released on July 17th (phase A) and July 18th (phase B) and the third on August 7th (phase A) and August 8th (phase B). As in the case of Task 1a, the participants had 21 hours to upload their results.

### Results in Task 1a

According to [34] the appropriate way to compare multiple classification systems over multiple datasets is based on their average rank across all the datasets. On each dataset the system with the best performance gets rank 1.0, the second best rank 2.0 and so on. In the case that two or more systems tie, they all receive the average rank.

Table 6 presents the average rank (according to MiF and LCaF) of each system over all the test sets for the corresponding batches. For comparison, the MTIFL, MTI and bioasq_baseline baseline systems used throughout the challenge are shown as well. MTIFL and MTI refer to the NLM Medical Text Indexer system [35]. Note, that the average ranks are calculated for the 4 best results of each system in the batch according to the rules of the challenge [36]. The best ranked system is highlighted with bold typeface. We can observe that during the first batch the MTIFL baseline achieved the best performance in terms of the MiF measure, but was matched to the performance of the RMAIP system in terms of the LCaF measure. Interestingly, the ranking of the RMAIP according to the LCaF measure is better than the one based on its MiF performance, which shows that RMAIP is able to give answers in the neighborhood (as designated by the hierarchical relations among the classes) of the correct ones. Overall, the MTIFL performed best in the first batch, with RMAIP and system3 following close.

**Table 6:** **Average ranks for each system across the batches of Task 1a for the measures MiF and LCaF**

**System**	**Batch 1**		**Batch 2**		**Batch 3**	
	**MiF**	**LCaF**	**MiF**	**LCaF**	**MiF**	**LCaF**
MTIFL	**1.25**	**1.75**	2.75	2.75	4.0	4.0
system3	2.75	2.75	**1.0**	**1.0**	2.0	2.0
system2	-	-	1.75	2.0	3.0	3.0
system1	-	-	-	-	**1.0**	**1.0**
MTI	-	-	-	-	3.25	3.0
RMAIP	2.50	**1.75**	5.0	4.5	5.25	5.5
RMAI	3.25	3.0	5.0	4.5	8.5	7.25
RMAIR	6.25	6.0	4.5	3.25	6.25	6.25
RMAIA	5.75	5.5	4.0	5.25	7.25	5.75
RMAIN	4.50	3.25	6.0	5.0	6.5	6.25
Wishart-S3-NP	8.75	9.0	14.25	15.0	-	-
Wishart-S1-KNN	8.75	9.25	12.25	12.5	-	-
Wishart-S5-Ensemble	9.5	8.0	9.50	10.25	-	-
mc4	14.75	14.25	21.0	21.0	21.5	21.25
mc3	11.0	11.25	19.75	19.75	22.0	21.5
mc5	11.25	10.0	15.0	14.75	17.0	17.0
cole_hce2	9.25	9.5	11.25	9.25	12.75	12.0
bioasq_baseline	14.0	14.0	17.75	16.75	20.75	
cole_hce1	13.5	13.5	14.75	14.0	16.0	14.75
mc1	8.75	8.25	13.75	13.25	13.0	13.5
mc2	11.25	11.5	17.75	18.25	14.25	15.75
utai_rebayct	15.5	16.0	16.75	17.5	19.25	21.5
Wishart-S2-IR	9.75	10.75	8.5	9.25	-	-
Wishart-S5-Ngram	-	-	10.5	9.75	-	-
utai_rebayct_2	-	-	-	-	18.25	18.5
TCAM-S1	-	-	-	-	11.25	12.25
TCAM-S2	-	-	-	-	12.25	12.25
TCAM-S3	-	-	-	-	12.5	12.5
TCAM-S4	-	-	-	-	12.0	12.75
TCAM-S5	-	-	-	-	12.75	12.0
FU_System	-	-	-	-	24.0	23.25

A hyphenation symbol (-) is used whenever the system participated in less than 4 times in the batch. The 4 best runs in each batch for each system were considered for its ranking.

In the other two batches the systems proposed in [32] ranked as the best performing ones occupying the first two places (system3 and system2 for the second batch and system1 and system 2 for the third batch). These systems follow a simple machine learning approach which uses SVMs and the problem is treated as flat. We note here the good performance of the *learning to rank* systems (RMAI, RMAIP, RMAIR, RMAIN, RMAIA). *Learning to rank* methods are mostly used in information retrieval tasks for ranking the retrieved results. Typically such methods make use of supervised machine learning techniques in order to construct ranking models for the purposes of retrieving documents. The aim of these models is to produce a permutated list of the ranked results that fits as much as possible the ranking observed in the training data.

According to the available descriptions, the only systems that made use of the MeSH hierarchy were the ones introduced by [37]. The top-down hierarchical systems, cole_hce1 and cole_hce2, achieved mediocre results while the utai_rebayct systems had poor performances. For the systems based on a Bayesian network, this behavior was expected as they cannot scale well to large problems. On the other hand the question that arises is whether the use of the MeSH hierarchy can be helpful for classification systems as the labels that are assigned by the curators to the PubMed articles do not follow the rule of the most specialized label. That is, an article may have been assigned a specific label in a deeper level of the hierarchy and at the same time a label in the upper hierarchy that is ancestor of the most specific one. In this case the system that predicted the more specific label will be penalized by the flat evaluation measures for not predicting the most general label, which is implied by the hierarchical relations.

### Results in Task 1b

#### Phase A

As in Task 1a the evaluation included three test batches. For phase A of Task 1b the systems were allowed to submit responses to any of the corresponding categories, that is documents, concepts, snippets and RDF triples. For each category, we ranked the systems according to the Mean Average Precision (MAP) measure [38]. The final ranking for each batch is calculated as the average of the individual rankings in the different categories. The detailed results for Task 1b phase A can be found in http://bioasq.lip6.fr/results/1b/phaseA/.

Table 7 presents the average ranking of each system in each batch of Task 1b phase A. It is evident from the results that the participating systems did not manage to perform better than the two baselines that were used in phase A. Note also that the systems did not respond to all the categories. For example, the MCTeam systems did not submit snippets throughout the task. Focusing on the specific categories, like concepts, for the Wishart system we observe that it achieves to have a balanced behavior with respect to the baselines (Table 8). This is evident from the F-measure which is superior to the values of the two baselines. This can be explained by the fact that the Wishart-S1 system responded with short lists while the baselines returned always long lists (50 and 100 items respectively). Similar observations hold also for the other two batches.

**Table 7:** **Average ranks for each system for each batch of phase A of Task 1b**

**System**	**Batch 1**	**Batch 2**	**Batch 3**
Top 100 Baseline	1.0	**1.875**	**1.25**
Top 50 Baseline	2.5	2.375	1.75
MCTeamMM	3.625	4.5	3.5
MCTeamMM10	3.625	4.5	3.5
Wishart-S1	4.25	3.875	-
Wishart-S2	-	4.125	-

The MAP measure was used to rank the systems. A hyphen (symbol -) is used whenever the system did not participate in the corresponding batch.

**Table 8:** **Results for batch 1 for concepts in phase A of Task1b**

**System**	**Mean**	**Mean**	**Mean**	**MAP**	**GMAP**
	**precision**	**recall**	**F-measure**		
Top 100 Baseline	0.080	0.858	0.123	0.472	0.275
Top 50 Baseline	0.121	0.759	0.172	0.458	0.203
Wishart-S1	0.464	0.429	0.366	0.342	0.063
MCTeamMM	0.000	0.000	0.000	0.000	0.000
MCTeamMM10	0.000	0.000	0.000	0.000	0.000

#### Phase B

In phase B of Task 1b the systems were asked to report exact and ideal answers. The systems were ranked according to the manual evaluation of ideal answers by the BioASQ experts [38]. For reasons of completeness, we report also the results of the systems for the exact answers. To do so, we average the individual rankings of the systems for the different types of questions, that is Yes/No, factoids and list.

Table 9 presents the average ranks for each system for the exact answers. In this phase we note that the Wishart system was able to outperform the BioASQ baselines. Table 10 presents the average scores^d^ of the biomedical experts for each system across the batches. Note that the scores are between 1 and 5 and the higher it is the better the performance. According to the results, the systems were able to provide comprehensible answers, and in some cases like in the second batch, highly readable ones. For example Table 11 presents the answer of the Wishart-S1 system along with the golden answer to the question: *Which drug should be used as an antidote in benzodiazepine overdose?* Of course the quality of the answer depends on the difficulty of the question. This seems to be the case in the last batch where the average scores are lower with respect to the other batches. Also, the calculated measures using ROUGE seem to be consistent with the manual scores in the first two batches while the situation is inverted in the third batch.

**Table 9:** **Average ranks for each system and each batch of phase B of Task 1b, for the “exact” answers**

**System**	**Batch 1**	**Batch 2**	**Batch 3**
Wishart-S1	**2.0**	**1.0**	-
Wishart-S2	**2.0**	-	-
Wishart-S3	**2.0**	-	-
Baseline1	4.66	**2.33**	**2.33**
Baseline2	4.33	4.0	2.66
main system	6.0	4.33	3.0
system 2	-	5.33	3.33
system 3	-	5.5	3.66
system 4	-	5.5	-

The final rank is calculated across the individual ranks of the systems for the different types of questions. A dash symbol (-) is used whenever the system did not participate to the corresponding batch.

**Table 10:** **Average scores for each system and each batch of phase B of Task 1b for the “ideal” answers**

**System**	**Batch 1**	**Batch 2**	**Batch 3**
Wishart-S1	**3.95**	**4.23**	-
Wishart-S2	**3.95**	-	-
Wishart-S3	**3.95**	-	-
Baseline1	2.86	3.02	**3.19**
Baseline2	2.73	2.87	3.17
main system	3.35	3.39	3.13
system 2	-	3.34	3.07
system 3	-	3.34	2.98
system 4	-	3.34	-

The final score is calculated as the average of the individual scores of the systems for the different evaluation criteria. A hyphenation symbol (-) is used whenever the system did not participate in the corresponding batch. The scores are given by experts who read and evaluated the “ideal” answers, and they range from 1 to 5, with 5 being the best score.

**Table 11:** **The “ideal” answers returned from the system Wishart-S1 along with the golden one**

**Wishart-S1**	**Golden answer**
Benzodiazepine (BZD) overdose (OD) continues to cause significant morbidity and mortality in the UK. Flumazenil is an effective antidote but there is a risk of seizures, particularly in those who have co-ingested tricyclic antidepressants. (PMID: 21785147) Flumazenil is a benzodiazepine antagonist. It is widely used as an antidote in comatose patients suspected of having ingested a benzodiazepine overdose. (PMID: 19500521)	Flumazenil should be used in all patients presenting with suspected benzodiazepine overdose. Flumazenil is a potent benzodiazepine receptor antagonist that competitively blocks the central effects of benzodiazepines and reverses behavioral, neurologic, and electrophysiologic effects of benzodiazepine overdose. Clinical efficacy and safety of flumazenil in treatment of benzodiazepine overdose has been confirmed in a number of rigorous clinical trials. In addition, flumazenil is also useful to reverse benzodiazepine induced sedation and to diagnose benzodiazepine overdose.

### Discussion of results

Task 1a ran for 18 weeks with a large number of documents (88,628) provided for testing. A total of 12 teams from various countries in three continents (Europe, North America and Asia) participated, representing academic (e.g., University of Alberta in Canada and Aristotle University of Thessaloniki in Greece), as well as industrial research (e.g., Toyota Technological Institute in Japan and Mayo Clinic in the USA). Competition was particularly intense, with each team participating with more than one systems (up to 5 were allowed). The MTI system of NLM was used as one of the baseline systems and was particularly hard to beat, as it is used to recommend MESH terms to the MEDLINE curators, in order to speed up their work. The evaluation of the systems was based on both established measures used for flat classification, as well as novel hierarchical measures, proposed by the BIOASQ consortium. Separate winners were announced for each batch and were awarded the corresponding prizes. The winning teams used various advanced text mining techniques and the positive surprise was that one of the systems [32] consistently outperformed the highly optimised MTI baseline. This finding suggests that there is still room of improvement for the systems used to suggest MESH headings to the professional indexers.

With regards to Task 1b, due to its complexity, both participation and evaluation of the results was particularly demanding. Three teams with long experience and infrastructure in question answering participated in the task, representing again both academic (University of Alberta in Canada) and industrial research (Toyota Technological Institute in Japan and Mayo Clinic in the USA). Automated evaluation of the results were provided for all aspects of the challenge, including intermediate and final results e.g., mean average precision in Phase A; accuracy, mean reciprocal rank, mean F-measure for exact answers to yes/no, factoid, and list questions in Phase B; ROUGE for ideal answers. However, in addition to the automated scores, the BIOASQ biomedical expert team was asked to provide manual scores (for readability, information recall and precision, lack of repetitions) on the final “ideal” answer that each system produced in Phase B. Despite the complexity of the task and the short time that the participants had for preparing their systems, the BIOASQ experts seemed particularly satisfied about the result that the participants produced, judging from the manual scores that they provided. Overall, the results of Task 1b suggest that there are already existing technologies that can address biomedical question answering in a manner which can be judged as satisfactory by experts. However, judging from the results in both phases of Task 1b, we can conclude that the task is far from being solved; a first important step was made though, through the BIOASQ competition and its challenges.

### Evaluation of the challenge and lessons learnt

In an effort to evaluate the BIOASQ challenge, we designed quantitative and qualitative measures. The quantitative evaluation was conducted by measuring the number of participants both in the challenge and the workshop that the results were presented, the number of visitors in our websites, and the downloads of the benchmark data. For the qualitative evaluation, we used questionnaires distributed to the participants of the challenge and the workshop and to the team of biomedical experts. As a summary, the analysis of the evaluation showed satisfactory results. The participation was satisfactory, especially in Task 1A, and most of the participants have already expressed interest to participate in the next cycles of BIOASQ, but they also intend to recommend it to other research groups. Finally, we had a very good cooperation with the team of biomedical experts, providing them with all the help and tools they needed for the creation of the benchmark datasets for Task 1B and the evaluation of the systems’ responses. The complete evaluation of the challenge, including the distributed questionnaires and the analysis of responses can be found in Deliverable D5.2 of the BIOASQ project [39].^e^ In the remaining of the section we present the highlights and discuss the main problems faced.

With regards to the quantitative measures’ results, there were 117 users registered on the BIOASQ’s participants area, with a total of 697 benchmark dataset downloads. In total, 11 different teams participated in the BIOASQ challenge. Given that one important objective of the challenge is to establish BIOASQ as a reference point for the biomedical community, these statistics constitute a good basis that indicates that the challenge is in the right direction.

The qualitative evaluation was conducted using questionnaires distributed to the participants and to the biomedical experts that created the benchmark questions and answers. The questionnaires distributed to the participating teams included questions targeting several aspects of the individual tasks, like the quality of the datasets, the technical support etc. Additionally, more general questions were provided to capture the overall impression of the participants for the BIOASQ challenge. The analysis of the questionnaires from the participants showed that they were satisfied with the challenge and as a consequence, not only are they willing to participate in the next cycle of the challenge, but they are willing to recommend BIOASQ to other research groups as well. However, there were some difficulties in the general understanding of the challenge. To alleviate this problem we modified the guidelines of the challenge for both tasks in order to make them clearer for the potential participants. Another problem we had to face was the low participation in Task 1b. For that we came up with two strategies. The first one was to identify all the research areas that relate to BIOASQ and modify the first page of our official website to include this information. The second one was to create a list of potential participants and invite them, through personal contact, to participate in the challenge. We hypothesize that in any future effort these problems should be addressed from the beginning of the challenge, in order to make the challenge even more attractive.

As far as the interaction with the team of the biomedical experts is concerned, the major problem that we faced pertained to the coordination of the experts’ team and the provision of tools and technical support that would help them in the creation of the benchmarks. After the end of the first cycle of the challenge, we distributed questionnaires to the biomedical experts, in order to assess the quality of the tools and their interaction with the organizing team. The analysis of the answers to the questionnaires showed that the experts were satisfied by the tools, and they are also willing to use them again in the future and even recommend them to others. Particularly for the annotation tool they are willing to use it for their own work. Overall, both the quantitative and the qualitative analysis show that the BIOASQ challenge was successful, leaving very good impression to the participants and the biomedical experts that contributed to the creation of the benchmarks.

## Conclusions

In this paper we have presented the background, the organisation and the results of the two tasks within the 2013 BIOASQ challenge. The tasks included in the BIOASQ challenge helped advance the state of the art in two fields. First, the automatic classification of biomedical documents using concepts from knowledge bases such as the Medical Subject Headings (MESH). In this task systems were required to tag large numbers of scientific biomedical articles with terms from a predefined biomedical vocabulary. Second, the automated question answering in the biomedical domain. In this task the systems were evaluated on how well they could identify text fragments in scientific articles, and related data in public knowledge bases, in order to answer questions set by the biomedical expert team of BIOASQ. In order to support the challenge, BIOASQ has built powerful and agile infrastructure for developing benchmark data sets for biomedical semantic indexing and question answering, as well as for using these data to evaluate participating systems, either automatically or manually. Most of the software produced by BIOASQ is provided as open source and the data are provided free of charge for future research use. The basic tool provided for benchmark data generation is the BIOASQ annotation tool, which can be used by biomedical experts to create questions and answering material of the form used in task 1b of the first BIOASQ challenge. The tool is publicly available as an open-source project at https://github.com/AKSW/BioASQ-AT. In addition to the tools provided for the biomedical experts, BIOASQ has constructed a platform for setting up and managing the evaluation campaign. The platform is available online at http://bioasq.lip6.fr/. The functionality of the online platform includes: (i) the unit that enables users to register in the platform, (ii) the Web services and the Web interface that enable users to upload/download data, (iii) the evaluation function that calculates automatically the evaluation measures, (iv) the discussion forum, (v) the detailed online documentation and guidelines for both tasks, and, (vi) an e-mail help desk that is publicly accessible. The source code of the platform will be made openly available at the end of the project, in order to be used in the future to set up new biomedical QA challenges, possibly based on new benchmarks produced by the BIOASQ expert network and/or with additional challenge tasks.

With regards to the challenge results and the BIOASQ’s expected impact, the main long-term goal of BIOASQ is to push significantly the research in information systems and methods that aim in turn at improved access to biomedical information. The potential impact of such a development is enormous and affects the biomedical experts, companies providing services in this industry, including information technology providers, and eventually everyone who will benefit from improved biomedical processes. On the way to this big goal, the first BIOASQ challenge facilitated a number of significant intermediate results. Primarily it facilitated a better understanding of the current semantic indexing and question answering technologies and their limitations. In addition, it enabled improved awareness of the biomedical community about the possibility of significant improvement of their work, using intelligent information systems. Furthermore, it established bridges among information technologists and biomedical experts, with the common goal of creating challenging tasks for current information systems. Last but not least, BIOASQ created a number of tools, infrastructure and benchmark data that facilitate the organisation of BIOASQ challenges, beyond the end of the project, and provide a very good basis for future research work in the fields of biomedical semantic indexing and question answering.

## Methods

### Formation of the BIOASQ biomedical experts team

The biomedical expert team was established during the first two months of the BIOASQ project. Several experts had been considered from a variety of institutions across Europe. The final selection of ten experts was based on the need to cover the broad biomedical scientific field, representing as much as possible, medicine, biosciences and bioinformatics. All the members of the biomedical team hold senior positions in universities, hospitals or research institutes in Europe. Their primary fields of research interests are the following: cardiovascular endocrinology, psychiatry, psychophysiology, pharmacology, drug repositioning, cardiac remodelling, cardiovascular pharmacology, computational genomics, pharmacogenomics, comparative genomics, molecular evolution, proteomics, mass-spectometry, and protein evolution.

The principal task of the biomedical expert team is the composition of the Question/Answer benchmark dataset which is used during the BIOASQ challenge Task 1b. Moreover, it is envisaged to be further enriched in the future mainly by voluntary contributions from the community. In this direction, the BIOASQ social network is expected to serve that purpose. For the first year, the benchmark dataset contains 29 development and 282 test questions used in a dry-run test and in the challenge respectively, along with gold standard (reference) answers. For each benchmark question composed by a biomedical expert, relevant material is also provided. This includes: (a) documents from specific article and abstract repositories, (b) concepts from designated ontologies, and, (c) statements (RDF triples), from selected life science triple stores. Next, text snippets that include information relevant to the composition of the answer are extracted from the selected documents. The experts have been trained to the needs of the annotation task during three physical meetings and by means of specifically composed guidelines. In these meetings, opinions were exchanged between the experts and members of the BIOASQ consortium, which further contributed to the optimization of the finally adopted QA composition methodology. In total, 500 questions are expected to be composed for the needs of the second year challenge, corresponding to approximately 50 QAs per expert. The members of the team are also assigned the task of the manual assessment of the responses provided by the competitors. During this step, they were given the opportunity to modify the QA gold standard by incorporating original data from the material returned by the participants. The bioexperts also contribute to the overall challenge evaluation through questionnaires and take part in the composition of the BIOASQ roadmap.

### Selection of data sources

For the task 1b the BIOASQ challenge aimed to cover a wide range of biomedical concepts, through the use of ontologies and linked data that describe several facets of the domain. The selection of resources for these tasks follows the triangle *drug-target-disease* which defines the prime information axes for any medical investigation. This *“knowledge-triangle”* supports the conceptual linking of biomedical knowledge databases and the processing of the related resources. The idea behind the drug-target-disease triangle is based on the tight links that exist between these three entities. Any disease is associated with research on prognosis, diagnosis, symptoms, co-morbidities and incidence/prevalence. Towards investigating these aspects, there is a tight relation between a disease and targets, e.g., with regards to the development of animal models or the research in diagnostics, that can help the researchers understand better the disease. The research on targets play a crucial role in turn for the study of sensitivity, selectivity, mutations of genes and biological functions that are connected to the disease. In parallel, the study of related drugs is crucial, because it can provide information on the pathways, mechanisms of action, efficacy and dosing, as well as for the design of clinical guidelines and clinical trials that aim at providing therapies to the disease. Hence, these three entities are tightly connected, and their relation has also constituted the basis for research on how automated systems may support the design of clinical trial protocols and the validation of hypotheses, e.g., as was the case of the *PONTE* European project [40]. From this perspective, the selected resources were the following: *Jochem* for drugs, GENE ONTOLOGY and UNIPROT for targets, DISEASE ONTOLOGY for diseases, MESH as a general purpose domain dictionary, PUBMED and PUBMED CENTRAL for documents, and LINKED LIFE DATA for triples (statements). An analytical description of the selected resources, and a more detailed discussion on the selection process can be found in Appendix B.

### The BIOASQ annotation tool

The annotation tool was developed to support the creation of the benchmark data for the challenge Task 1b. It was specifically designed to enable the biomedical experts to create the gold standards for these tasks. In its current version, the tool enables its users to:

- create evaluation questions or continue working on existing questions,
- search for relevant concepts, documents and triples that allow answering the questions,
- associate the evaluation questions with gold standard answers,
- annotate them with concepts from designated taxonomies or ontologies, and,
- associate the answers with relevant triples and snippets from selected data sources.

The tool is publicly available [41], and in the following, the basic functionalities of the tool are explained.

**Figure 4:**
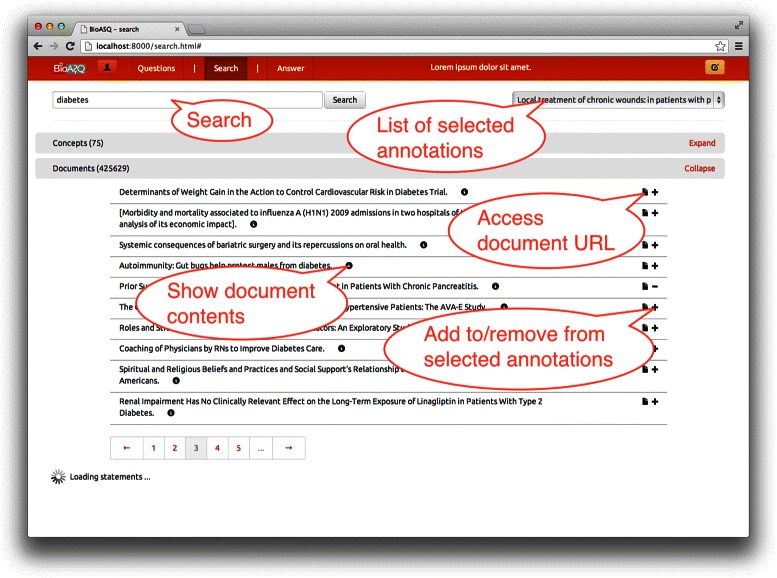
Screenshot of the annotation tool’s search and data selection screen with the section for document results expanded. The search interface accepts a number of keywords that are sent in parallel to each of the GOPUBMED services. Upon retrieval of the last response, results are combined and returned to the frontend. The client creates one request for each of the result domains (concepts, documents, statements). Whenever results are retrieved for a domain, the respective section of the *GUI* is updated immediately. Each search result displays the title of the result.

As a first step, the users of the annotation tool may login and either create (formulate) a new question in which they want to start working on, or continue working from an existing question that has been stored from previous sessions. The main screen of the annotation tool is shown in Figure 4, where at the top the user can navigate among the three main functionalities using the respective tabs: (1) create a new question, or select to continue working on an existing one (Questions tab), (2) search for concepts, documents and triples for the selected question (Search tab), and, (3) annotate relevant snippets from the selected documents and formulate and store the final answer to the question (Answer tab).

In Figure 4 the search tab is selected and the user can type a query to the search bar (top left) and retrieve relevant concepts, documents and triples from the used *BioASQ* resources. Any of the results can be added to the list of the related items for the question, by using the plus symbol, and an existing one can be removed with the minus symbol. If there is any additional information for any returned item, e.g., the full text of a returned document, or the definition page of an ontology concept, this is accessible directly via the tool. For the ranking and ordering of the retrieved results, standard *TF-IDF* (Term Frequency-Inverse Document Frequency) scoring is used for the document terms, and the ontology concepts. For the triple search, the triple store is indexed in a *Lucene* index, and *TF-IDF* is also used for the ranking.

**Figure 5:**
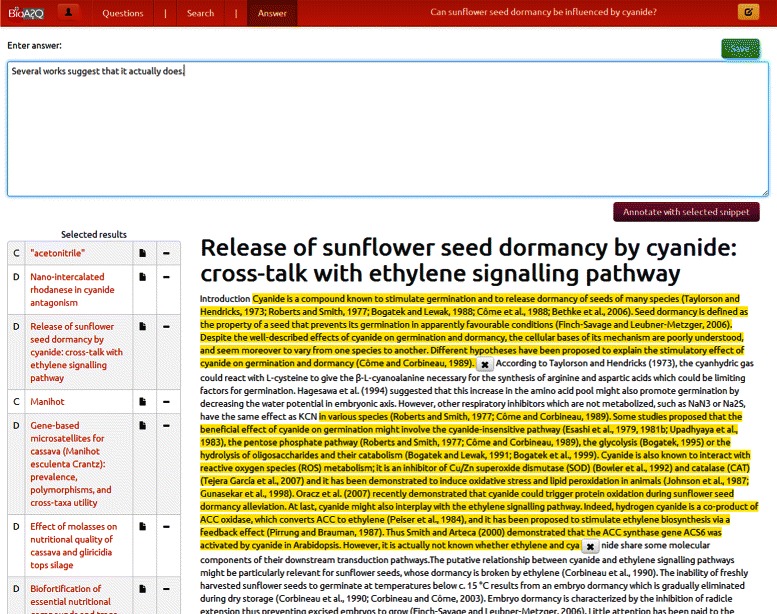
Screenshot of the answer formulation and annotation with document snippets. The process of formulating the answer to the selected question and its annotation with document snippets by the domain expert is shown. The user can either dismiss items that were selected in the previous step, or add snippets (i.e., document fragments) as annotations to the answer.

Finally, as illustrated in Figure 5, once the user navigates to the Answer tab, he can formulate the answer to the question. In this case the question is shown at the top right: *“Can sunflower seed dormancy be influenced by cyanide?”*. Any of the selected documents, concepts or triples from the previous step (Search screen) are listed here in the left of the user’s screen. Besides typing the ideal answer (top of the screen), the user can also click on selected documents (left of the screen) to annotate snippets from these documents that are relevant and important for the formulation of the answer (middle of the screen). The process is considered completed when the user clicks on the *“Save”* button (top right of the screen), in which case, both the answer to the question and all of the associated annotations are stored in a back-end database. This allows the users to continue working on any questions they have started in any previous sessions. The data storage implementation of the BIOASQ annotation tool relies on the *NoSQL* database *MongoDB* [42]. To aid the experts towards using the tool, a set of guidelines for producing the benchmark questions was distributed to them [43], which can also be used as general guidelines for future usage of the tool. The guidelines give clear examples of how to use the annotation tool, what to avoid when searching, and when formulating the questions, and how to make the best use of the tool, i.e., in terms of familiarizing the experts with the functionality and the behavior of the tool.

### The BIOASQ participants area and evaluation platform

The series of the BIOASQ challenges require frequent exchange of data between the challenge participants and the organisers. The developed machanisms that cover those needs are integrated in an online platform^f^, the BIOASQ *Participants Area*. The BIOASQ *Participants Area* provides mechanisms for the participants to find information and support regarding the challenge and participate in the tasks. On the other hand, the BIOASQ team using the platform can administrate the challenge, release the benchmark datasets and provide the necessary mechanisms that will allow the evaluation of the participating systems.

The platform was designed to be user-friendly. A key concept was to make it simple enough so that participants would not need much time to exchange data, receive support or check the performance of their systems by browsing the evaluation measures. Another key concept was to provide ways to automate the process of downloading and submitting results. For this reason, web services were developed so that users could programmatically download the test sets and submit results saving time and effort.

The platform provides a set of mechanisms to the registered users of the challenge. After subscribing to the platform, they gain access to the following:

- the BioASQ benchmark datasets; they consist of training and test datasets which are available for downloading after their release,
- detailed guidelines describing the BioASQ tasks,
- tools that have been developed to help participants process the datasets e.g. tokenizers for Task 1a,
- mechanisms for submitting results; they include:
  - HTML forms available as long as there are active tests, and
  - Web services for submitting results in an automated way,
- tables for browsing the evaluation results,
- the *“BioASQ Discussions Area”*, which is a forum about the BioASQ challenge, and
- an e-mail help desk for contacting the organising team.

### Description of Task 1a systems

The participating systems in the semantic indexing task of the BIOASQ challenge adopted a variety of approaches, like hierarchical and flat methods or search-based systems that rely on information retrieval techniques. In the rest of this section we describe the proposed systems by focusing on their key points.

In [37] the authors proposed two hierarchical approaches (participating systems cole_hce1 and cole_hce2). The first approach, referred to as *Hierarchical Annotation and Categorization Engine* (HACE), follows a top-down hierarchical classification scheme [3] where, for each node of the hierarchy, a binary classifier is trained. For constructing the positive training examples for each node, the authors employ a random method that selects a fixed number of examples from the descendants of the current node and a method that is based on *k*-means to choose the *k* closest examples to the centroid of the node. In both approaches the selected examples are fixed in order to create manageable datasets especially in the upper levels of the hierarchy. The second system (*Rebayct*) that has participated in the challenge was based on a Bayesian network which models the hierarchical relations as well as the training data (that is the terms in the abstracts and titles). A major drawback of this system is that it cannot scale well to large classification problems with thousands of classes and millions of documents. For this reason, the authors reduced drastically the training data keeping only 10*%* of the data split in 5 disjoint parts in order to train five different models. During the testing phase, the models are aggregated through a simple majority voting.

In [32] (*AUTH*) a flat classification approach has been employed (participating systems system1-5) which trains a binary SVM for each label in the training data [44]. In order to reduce the complexity of the problem the authors kept only the training data that belong to the journals (1806 in total) from which the test sets were sampled during the testing phase of the challenge. The journal filtering reduced the training data to approximately 4 millions of documents from the initial 11 millions documents. The features that were used to represent each article were unigrams and bigrams (word as unit) extracted from the title and abstract of each article. The systems that were introduced in the challenge use a meta-model (called MetaLabeler [44]) for predicting the number of labels (*N*) of a test instance. During the prediction all the SVM classifiers are queried and the labels are sorted according to the corresponding confidence value. Finally, the system predicts the *N* top labels. While the proposed approach is relative simple, it requires processing power for both the training and the testing procedure. Furthermore, it has large storage requirements (as reported from the authors, the size of the models for one of the systems was 406Gb).

In [45], the authors follow two different approaches (participating systems mc1-5): (a) one that relies in the results provided by the MetaMap tool described in [46], and, (b) one that is based on the search engine Indri [47]. In the MetaMap based approach, for each test instance, the MetaMap system is queried for both the title and the abstract of the article. The returned results contain concepts and their corresponding confidence scores. The system calculates a final score, weighting differently the concepts that are obtained for the title and the abstract and filtering the ones exceeding a predefined threshold for the confidence score. Finally, the system proposes the *m* top-ranked concepts, where *m* is a free parameter. In the search based approach the authors index the training data using the engine Indri. For each test article a query *q* is formed and a score is calculated for each document *d* in the index. The concepts of the *m* top-ranked documents are assigned to the test article.

In the *Wishart* system [48] a typical flat classification approach as well as a *k*-NN are used. In the flat approach, a binary SVM is trained for each label present in the training data using as features unigrams, bigrams and trigrams extracted from the abstracts of the training data. In the *k*-NN based approach, for each test article, the *k*-NN method is invoked in order to retrieve documents from a local index. Additionally, the NCBI Entrez system is queried in order to retrieve extra documents along with their labels. All the abstracts are ordered (first *N* - empirically set to 100) according to their distance and the top *M* (empirically set to 10) labels are retained. For the final prediction, the two systems are combined by keeping the common predicted labels; the other labels are ordered according to their confidence scores. The system predicts 10-15 labels for each test article.

A learning to rank method has been used in the NCBI team [49]. More specifically, the systems (all RMAI participating systems) follow a three stage approach: (i) first the *k*-nearest neighbours of the test article are retrieved from the MEDLINE database, (ii) next the labels are ordered using a learning to rank algorithm and (iii) finally a cut-off method prunes the ordered list. It is interesting to note that in the definition of the features for the learning to rank problem, the authors use the results of the MTIFL baseline system (see next paragraph). More specifically, a binary feature indicates whether a specific label is observed in the results of MTIFL.

Table 12 summarizes the main technologies that were employed by the participating systems; it also indicates whether a hierarchical or a flat approach has been followed. Additionally, the last column shows what features were used from each team for the representation of the documents. It is clear that the majority of the participants followed flat methods to tackle the problem using a variety of technologies from the machine learning and information retrieval areas. Not surprisingly, the machine learning approaches used SVM classifiers which are powerful schemes in text classification tasks [32,48]. On the contrary, these flat systems have large processing and storage requirements in both training and inference stages. In order to reduce the complexity of the problem in [37], the authors leveraged the hierarchy information by employing the classifiers in a top-down manner. In [45] and [49] the authors follow a two stage approach, thus reducing the complexity, where they first retrieve relevant articles using search engines or following a k-nearest neighbors approach on local indexes of the training data.

**Table 12:** **Technologies used in Task 1a from the participating systems along with the feature representation of the documents**

**Reference**	**Approach**	**Technologies**	**Features**
[32]	flat	SVMs, MetaLabeler [44]	unigrams, bigrams
[37]	hierarchical	SVMs, Bayes networks	unigrams, bigrams
[45]	flat	MetaMap [46],	unigrams
		information retrieval,	
		search engines	
[48]	flat	k-NN, SVMs	unigrams, bigrams,
			trigrams
[49]	flat	k-NN, learning-to-rank	unigrams

Unigrams, bigrams and trigrams refer to the word level.

### Description of Task 1b systems

In the second task of the BIOASQ challenge a total of three teams participated in both phases with 11 systems. Only two descriptions were available from these systems [45,48].

For phase A of Task 1b the Wishart system [48] makes use of query processing and document ranking techniques. More specifically, each test question in natural language form is converted by extracting the noun phrases and referencing them using a thesaurus of biomedical entities. Then the question is expanded by adding synonyms and relevant biomedical entities using the PolySearch tool [50]. The entities found by PolySearch are used to rank the retrieved set of concepts, articles, triples and snippets. In phase B of the task a similar approach to phase A is used in order to augment the set of given concepts. Extracted sentences from the retrieved documents are ranked according to the cosine similarity with respect to the augmented concepts. The top-ranked sentences are concatenated in order to provide an *ideal* answer.

The MCTeam system participated [45] only in phase A. In order to form an appropriate query the system first uses the test question to query MetaMap, which responds with concept-related words. These words were used to form a query. In case where no concepts were returned by MetaMap, the final query was formed by removing the stopwords from the test question. This query was used to retrieve the appropriate information from the BIOASQ web services and also from a local index of PubMed full-text articles^g^. The two lists of the retrieved results were then merged and formed the final results.

## Availability of supporting data

All of the produced datasets in the framework of the BIOASQ challenge, and the results of all participating teams, are publicly available for download and usage via the BIOASQ participants area: http://bioasq.lip6.fr/. Further documentation on the organization of the BIOASQ challenge may be found at the official BIOASQ website: http://www.bioasq.org/.

## Endnotes

^a^ An in-house stop word list that is specific to the domain is used.

^b^ More info about the prizes can be found at: http://bioasq.org/participate/prizes

^c^ According to the rules of BioASQ, each system had to participate in at least 4 test sets of a batch in order to be eligible for the prizes.

^d^ Please consult the description of the evaluation measures used in the challenge for more information.

^e^ All of the project deliverables are publicly available at: http://bioasq.org/project/public_documents

^f^ Publicly available under http://bioasq.lip6.fr.

^g^ The Indri search engine has been used for indexing the documents.

^h^ AP approximates the area under a recall–precision curve; consult [51].

## Appendix A

### Evaluation measures for Task 1a

As the task concerns the classification in a hierarchical setting, flat evaluation measures (that ignore the presence of relations among the classes) are not sufficient for a proper evaluation of classification systems. A hierarchical measure can also be included that takes into account the relations in the given hierarchy (in our case MESH) and assigns a value accordingly. Thus, for the assessment of the systems participating in Task 1a, one flat and one hierarchical measure are used. Specifically, the flat micro-F1 measure is used which is a label-based measure [24]:

$$MiF1 = \frac{2*MiP*MiR}{MiP+MiR}, $$

where *MiP* and *MiR* are the micro-precision and micro-recall measures calculated as follows:

$$MiP = \frac{\sum_{i=1}^{|C|}tp_{c_{i}}}{\sum_{i=1}^{|C|}(tp_{c_{i}}+fp_{c_{i}})} $$

$$MiR = \frac{\sum_{i=1}^{|C|}tp_{c_{i}}}{\sum_{i=1}^{|C|}(tp_{c_{i}}+fn_{c_{i}})} $$

where $tp_{c_{i}}, fp_{c_{i}}$ and $fn_{c_{i}}$ are respectively the true positives, false positives and false negatives for class *c*_*i*_.

From the family of hierarchical measures the Lowest Common Ancestor - F measure (*LCaF*) [25] is used:

(1) $$ LCaF = \frac{2*LCaP*LCaR}{LCaP+LCaR}  $$

where the corresponding precision and recall measures (*LCaP* and *LCaR* respectively) are calculated as follows:

(2) $$ LCaP = \frac{|\hat{Y}_{aug} \cap Y_{aug}|}{|\hat{Y}_{aug}|}  $$

(3) $$ LCaR = \frac{|\hat{Y}_{aug} \cap Y_{aug}|}{|Y_{aug}|}  $$

where *Y*_*aug*_ and $\hat {Y}_{\textit {aug}}$ are augmented sets of the true and the predicted classes respectively, based on the hierarchical relations. Specifically, in the case of *LCaF* these sets are constructed as follows:

1. First for each class *y* in the set of true classes *Y* the lowest common ancestor with respect to the set of predicted classes $\hat {Y}$ is calculated:
  $$LCA(y,\hat{Y}) = \arg\min_{m}\gamma(m,y), $$
  where *γ*(*u*,*v*) denotes the distance between the nodes *u* and *v* in the graph.
2. Symmetrically, for each class in $\hat {Y}$ the $LCA(\hat {y},Y)$ is computed.
3. Then two graphs, *G*_*t*_ and *G*_*p*_, are defined containing the shortest paths from each *y*∈*Y* to $LCA(y,\hat {Y})$ for *G*_*t*_ and $\hat {y} \in \hat {Y}$ to $LCA(\hat {y},Y)$ for *G*_*p*_.
4. Finally, Equation 1 is applied to the sets of the nodes defined by the two graphs.

*LCaF* assigns the minimum cost and, thus, handles the over-penalization of errors that occurs in multi-label problems with *DAG* hierarchies. Though the details of the *LCaF* measure are presented in detail in [25] in Figure 2 we illustrate the basic concept of the measure. Given a hierarchy of concepts, in the figure the nodes surrounded by circles are the true classes, e.g., concepts that should annotate the input document, while the nodes surrounded by rectangles are the predicted classes, e.g., concepts that a system has predicted as true. *LCaF* ia based on the notion of adding all ancestors of the predicted (rectangles) and true (circles) classes. However, adding all the ancestors has the undesirable effect of over-penalizing errors that happen to nodes with many ancestors. Thus, *LCaF* uses the notion of the Lowest Common Ancestor to limit the addition of the ancestors. For the challenge results calculation *LCaF* is first computed per instance and then averaged over all instances.

Given the aforementioned descriptions, the winners of each batch were decided based on their performance in the Micro F-measure (MiF) from the family of flat measures, and the Lowest Common Ancestor F-measure (LCaF) from the family of hierarchical measures. For completeness, several other flat and hierarchical measures are reported for all participating systems [38], but which are not used for the selection of the winner.

#### Evaluation measures for Task 1b

##### Evaluation process and measures for Task 1b Phase A

In Phase A, the participants were provided with English questions *q*_1_,*q*_2_,*q*_3_,…,*q*_*n*_. For each question *q*_*i*_, each participating system was required to return: ***A list of relevant concepts*** *c*_*i*,1_,*c*_*i*,2_,*c*_*i*,3_,… from the designated terminologies and ontologies. The list should be ordered by decreasing confidence, i.e., *c*_*i*,1_ should be the concept that the system considers most relevant to the question *q*_*i*_, *c*_*i*,2_ should be the concept that the system considers to be the second most relevant etc. ***A list of relevant articles*** (documents) *d*_*i*,1_,*d*_*i*,2_,*d*_*i*,3_,… from the designated article repositories. Again, the list should be ordered by decreasing confidence, i.e., *d*_*i*,1_ should be the article that the system considers most relevant to the question, *d*_*i*,2_ should be the article that the system considers to be the second most relevant etc. ***A list of relevant text snippets*** *s*_*i*,1_,*s*_*i*,2_,*s*_*i*,3_,… from the returned articles. Again, the list should be ordered by decreasing confidence. Each snippet is represented by the unique identifier of the article it comes from and the offsets (character positions in the article) of the snippet’s beginning and end (offsets of the first and last characters). ***A list of relevant*** RDF ***triples*** *t*_*i*,1_,*t*_*i*,2_,*t*_*i*,3_,… from the designated ontologies. Again, the list should be ordered by decreasing confidence.

For each question *q*_*i*_, the BIOASQ team of biomedical experts has constructed the gold (correct) sets of concepts, articles, snippets, and triples. The biomedical experts also inspected the concepts, articles, snippets, and triples of each system in order to add to the corresponding golden sets any correct (relevant) items that they had missed, but the systems managed to retrieve.

For each system, the lists of returned concepts, articles, snippets, and triples of all the questions were evaluated using the *mean average precision* (MAP) measure, defined below, which is widely used in information retrieval to evaluate ranked lists of retrieved items. We also used the *geometric mean average precision* (GMAP), which places more emphasis on improvements in low performing queries (see [51] and [52]). For the sake of completeness, we also computed the *mean precision*, *mean recall*, and *mean F-measure* of each system, also defined below, but the official scores for Phase A were based on MAP.

Given a set of golden items (e.g., articles), and a set of items returned by a system (for a particular question in our case), precision (*P*) and recall (*R*) are defined as follows:

(4) $$ P = \frac{\mathit{TP}}{\mathit{TP} + \mathit{FP}}  $$

(5) $$ R = \frac{\mathit{TP}}{\mathit{TP} + \mathit{FN}}  $$

where *T* *P* (true positives) is the number of returned items that are also present in the golden set, *F* *P* (false positives) is the number of returned items that are not present in the golden set, and *F* *N* (false negatives) is the number of items of the golden set that were not returned by the system. The *F*_*β*_ measure is the weighted harmonic mean of *P* and *R*, defined as follows:

(6) $$ F_{\beta} = (1 + \beta^{2}) \cdot \frac{P \cdot R}{(\beta^{2} \cdot P) + R}  $$

For *β*=1, the same weight is assigned to both precision and recall, and the resulting measure, often called simply *F*-measure, is defined as follows:

(7) $$ F_{1} = 2 \cdot \frac{P \cdot R}{P + R}  $$

Given a set of queries (in our case, questions) *q*_1_,…,*q*_*n*_, the *mean precision*, *mean recall*, and *mean F-measure* of each system is obtained by averaging its precision, recall, and *F*-measure for all the queries.

In BIOASQ, we computed the mean precision, mean recall, and mean *F*-measure of the concepts, articles, snippets, and triples returned by each system. In the case of snippets, a complication is that a returned snippet may overlap with one or more golden snippets, without being identical to any of them. To take this into account, in the case of snippets we modify the definitions of precision and recall. Figure 3 illustrates what we mean by article-offset pairs. A snippet is determined by the article it comes from and by the offsets (positions) in the article of the first and last characters of the snippet. We can also think of the snippet as a set of (article, offset) pairs, one pair for each character of the snippet. In the example of Figure 3, Article 1 has *n* characters and a golden snippet starting at offset 3 and ending at offset 10. Let us call *S* the set of all the article-offset pairs of all the characters in the snippets returned by a system for a particular question, *G* the set of all the article-offset pairs of all the characters in the golden snippets of the question, and let |*s*| denote the cardinality of a set *s*. The definitions of precision (*P*_*snip*_) and recall (*R*_*snip*_) for snippets are:

(8) $$ P_{\mathit{snip}} = \frac{|S \cap G|}{|S|}  $$

(9) $$ R_{\mathit{snip}} = \frac{|S \cap G|}{|G|}  $$

In effect, *P*_*snip*_ divides the size (in characters) of the total overlap between the returned and golden snippets by the total size of the returned snippets, whereas *R*_*snip*_ divides the size of the total overlap by the total size of the golden snippets. The definitions of *F*_*β*_, mean precision, mean recall, and mean *F*-measure for snippets are the same as the corresponding definitions for concepts, articles, and triples, but they use *P*_*snip*_ and *R*_*snip*_ instead of *P* and *R*.

Precision, recall, and *F*-measure do not consider the order of the items returned by a system for each query. Recall that in BIOASQ we require the lists of concepts, articles, snippets, and triples that a system returns for each question to be ordered (ranked) by decreasing confidence. To take the ordering of a particular returned list (for a particular question) into account, it is common in information retrieval to compute the (non-interpolated) *average precision* (AP) of the list, defined as follows:

(10) $$ \textsc{AP} = \frac{\sum_{r=1}^{|L|} P(r) \cdot \mathit{rel}(r)} {|L_{R}|}  $$

where |*L*| is the number of items in the list, |*L*_*R*_| is the number of relevant items, *P*(*r*) is the precision when the returned list is treated as containing only its first *r* items, and *r* *e* *l*(*r*) equals 1 if the *r*-th item of the list is in the golden set (i.e., if the *r*-th item is relevant) and 0 otherwise.^8^ In BIOASQ, especially when computing the average precision of a list of *snippets*, *P*(*r*) is taken to be the snippet precision *P*_*snip*_ when the returned list of snippets is treated as containing only its first *r* snippets; and *r* *e* *l*(*r*) is taken to be 1 if the *r*-th returned snippet has a non-zero overlap (shares at least one article-offset pair) with at least one golden snippet of the particular question.

By averaging AP over a set of queries (in our case, questions) *q*_1_,…,*q*_*n*_, we obtain the *mean average precision* (MAP), defined as follows:

(11) $$ \textsc{MAP} = \frac{1}{n} \cdot \sum_{i=1}^{n} \textsc{AP}_{i}   $$

where AP_*i*_ is the average precision of the list returned for query (question) *q*_*i*_. In our case, each system received four MAP scores, for the lists of concepts, articles, snippets, and triples, respectively, that it returned for all the questions.

The *geometric mean average precision* (GMAP), defined below, is very similar to MAP, but it uses the geometric instead of the arithmetic mean, which places more emphasis on improvements in low performing queries, as already noted.

(12) $$ \textsc{GMAP} = \sqrt[n]{\prod_{i=1}^{n}(\textsc{AP}_{i} + \epsilon)}   $$

An alternative way to more easily compute GMAP is by using the following equation:

(13) $$ \textsc{GMAP} = \exp\left(\frac{1}{n} \cdot \sum_{i=1}^{n} \ln(\textsc{AP}_{i} +\epsilon)\right)   $$

In both versions of GMAP, *ε* is a small number added to handle cases where AP_*i*_=0. As with MAP, in BIOASQ each system receives four GMAP scores, for the lists of concepts, articles, snippets, and triples, respectively, that it returned for all the questions. The official scores for Task 1b Phase A were based on MAP, as already noted. Table 13 summarizes the evaluation measures of Phase A; the official measures are shown in bold.

**Table 13:** **Evaluation measures for Phase A of Task 1b**

**Retrieved**	**Unordered retrieval measures**	**Ordered retrieval**
**items**		**measures**
concepts	mean precision, recall, *F*-measure	**MAP**, GMAP
articles	mean precision, recall, *F*-measure	**MAP**, GMAP
snippets	mean precision, recall, *F*-measure	**MAP**, GMAP
triples	mean precision, recall, *F*-measure	**MAP**, GMAP

##### Evaluation process and measures for Task 1b Phase B

In Phase B, the participants were provided with the same questions *q*_1_,…,*q*_*n*_ as in Phase A, but this time they were also given the golden (correct) lists of concepts, articles, snippets, and triples of each question. For each question, each participating system returned an “ideal” answer, i.e., a paragraph-sized summary of relevant information. In the case of *“yes/no”*, *factoid*, and *list* questions, the systems also had to return “exact” answers; for summary questions, no “exact” answers were to be returned. The participants were told the type of each question.

We first discuss how “exact” answers were evaluated in Phase B, by considering in turn *“yes/no”*, *factoid*, and *list* questions. For each *“yes/no”* question, the “exact” answer of each participating system had to be either ‘yes’ or ‘no’. The response was compared against the golden “exact” answer (again ‘yes’ or ‘no’) that the BIOASQ team of biomedical experts has associated with the question. For each system, we computed the *accuracy* (ACC) of its responses to *“yes/no”* questions. Assuming that there are *n* *“yes/no”* questions, accuracy is defined as follows, where *c* is the number of correctly answered *“yes/no”* questions.

(14) $$ \textsc{ACC} = \frac{c}{n}  $$

For each *factoid* question, each participating system had to return a list of up to 5 entity names, ordered by decreasing confidence. The BIOASQ team of biomedical experts had associated with each *factoid* question a single golden entity name, as well as possible synonyms of that name.

We measured the *strict accuracy* (SACC) and *lenient accuracy* (LACC) of each system for *factoid* questions. Strict accuracy counts a question as correctly answered if the golden entity name (or a synonym of that name) is the first element of the list returned by the system. By contrast, lenient accuracy counts a question as correctly answered if the golden entity name (or synonym) is included, not necessarily as the first element, in the list returned by the system. In the definitions below, *n* is the number of *factoid* questions, *c*_1_ is the number of *factoid* questions that have been answered correctly when only the first element of each returned list is considered, and *c*_5_ is the number of *factoid* questions that have been answered correctly in the lenient sense, when all the elements of the returned list are considered.

(15) $$ \textsc{SACC} = \frac{c_{1}}{n}  $$

(16) $$ \textsc{LACC} = \frac{c_{5}}{n}  $$

Strict and lenient accuracy were measured for completeness. The official measure for the “exact” answers of factoid questions was the *mean reciprocal rank* (MRR), which is often used to evaluate *factoid* questions in question answering challenges; consult, for example, [8]. In the definition below, for each *factoid* question *q*_*i*_ we search the returned list looking for the topmost position that contains the golden entity name (or one of its synonyms). If the topmost position is the *j*-th one, then *r*(*i*)=*j*; otherwise *r*(*i*)→+*∞*, i.e., $\frac {1}{r(i)} = 0$.

(17) $$ \textsc{MRR} = \frac{1}{n} \cdot \sum_{i=1}^{n} \frac{1}{r(i)}  $$

In effect, MRR rewards systems that manage to include the golden responses (or their synonyms) higher in the returned lists.

For each list question, each participating system had to return a list of entity names, jointly taken to constitute a single answer (e.g., the most common symptoms of a disease). The BIOASQ team of biomedical experts had associated with each list question a golden list of entity names, also providing possible synonyms for each entity name of the golden list.

For each list question, the list returned by the system was compared against the golden list by computing its *precision* (*P*), *recall* (*R*), and *F-measure* (*F*_1_), as described earlier. Here *T* *P* is the number of entities that are mentioned both in the returned and the golden list; *F* *P* is the number of entities that are mentioned in the returned, but not in the golden list; and *F* *N* is the number of entities that are mentioned in the golden, but not in the returned list. If the same entity is mentioned using different synonyms in the returned and golden lists, it is counted as having been mentioned in both lists. If an entity is mentioned multiple times, possibly using different synonyms, in the returned list, it is counted only once.

By averaging precision, recall, and *F*-measure over the list questions, we obtained the *mean average precision*, *mean average recall*, and *mean average F-measure* score of each system for list questions. The official measure for list questions was mean *F*-measure. Table 14 summarizes the kinds of responses and the evaluation measures that were used in Phase B.

**Table 14:** **Evaluation measures for the “exact” answers in Phase B of Task 1b**

**Question**	**Participant**	**Evaluation measures**
**type**	**response**	
yes/no	yes or no	**accuracy**
factoid	up to 5 entity names	strict and lenient accuracy, ***MRR***
list	a list of entity names	**mean** precision, recall, ***F*** *-measure*

In the case of the “ideal” answers evaluation, the BIOASQ team of biomedical experts had associated each question with a golden “ideal” answer which can act as a reference for evaluating the systems’ “ideal” answers. The maximum allowed length of each “ideal” answer to be produced was set to be 200 words.

The “ideal” answers of the systems were evaluated both manually (by the BIOASQ team of biomedical experts) and automatically (by comparing them to the golden “ideal” answers). The official scores were based on the manual evaluation; the automatic evaluation was performed mostly to explore how well automatic evaluation measures (e.g., from multi-document text summarization) correlate with the scores of the biomedical experts.

For the manual evaluation, each one of the “ideal” answers of each system was inspected by a biomedical expert, who was asked to evaluate the answer in terms of *information recall* (the “ideal” answer reports all the necessary information), *information precision* (no irrelevant information is reported), *information repetition* (the “ideal” answer does not repeat the same information multiple times, e.g., when sentences of the “ideal” answer that have been extracted from different articles convey the same information), and *readability* (the “ideal” answer is easily readable and fluent). An 1−5 scale was used in all four criteria (1 for ‘very poor’, 5 for ‘excellent’). Table 15 summarizes the criteria that were used in the manual evaluation of the “ideal” answers in Phase B.

**Table 15:** **Criteria for the manual evaluation of the “ideal” answers in Phase B of Task 1b**

**Criterion**	**Explanation**	**Score**
information recall	All the necessary information is reported.	1–5
information precision	No irrelevant information is reported.	1–5
information repetition	The answer does not repeat the same information multiple times.	1–5
readability	The answer is easily readable and fluent.	1–5

The “ideal” answers returned by the systems were also automatically evaluated using ROUGE; consult [29]. Roughly speaking, ROUGE counts the overlap between an automatically constructed summary and a set of reference (golden) summaries constructed by humans. There are several different versions of ROUGE. ROUGEN, defined below, uses word *n*-grams when computing the overlap between an automatically constructed summary *S* and a set *R* *e* *f* *s* of reference summaries:

(18) $$\begin{array}{@{}rcl@{}} { \textsc{ROUGEN}}(S|\mathit{Refs}) = \frac{\sum_{R \in \mathit{Refs}} \sum_{g_{n} \in R} C(g_{n},S,R)} {\sum_{R \in \mathit{Refs}} \sum_{g_{n} \in R} C(g_{n},R)} \end{array} $$

In the definition above, *g*_*n*_ is a word *n*-gram, *C*(*g*_*n*_,*S*,*R*) is the number of times that *g*_*n*_ co-occurs in *S* and a reference summary *R*, and *C*(*g*_*n*_,*R*) is the number of times *g*_*n*_ occurs in reference *R*.

ROUGES uses skip bigrams, instead of *n*-grams, when computing the overlap. A skip bigram is any pair of words, maintaining the order of the two words and ignoring any intermediate words. ROUGESU is similar to ROUGES, but it also counts unigrams (individual words) that occur both in *S* and *R* *e* *f* *s*. The most widely used versions of ROUGE are R2 and RSU4, which have been found to correlate well with human judgements, when multiple reference summaries are available per question; consult [29]. R2 is ROUGEN with *n*=2; and RSU4 is a version of ROUGESU with the maximum distance between the words of any skip bigram limited to 4.

In BIOASQ, we used R2 and RSU4, with *S* being an “ideal” answer constructed by a system and *R* *e* *f* *s* being the golden “ideal” answer of the particular question *S* was constructed for. Table 16 summarizes the evaluation measures of Phase B; the official measures are shown in bold.

**Table 16:** **Evaluation measures for the “ideal” answers in Phase B of Task 1b**

**Question type**	**Participant response**	**Evaluation measures**
any	paragraph-sized text	R2, RSU4, **manual scores**

## Appendix B

### Detailed description of the selected data sources

In this appendix, the selected data sources (documents, databases, ontologies) that were used in the BIOASQ challenge are described. Ontologies such as the *Medical Subject Headings* (MESH) and the *Gene Ontology* (GENE ONTOLOGY) play a major role in biology and medicine since they facilitate data integration and the consistent exchange of information between different entities. They can also be used to index and annotate data and literature, thus enabling efficient search and analysis. In the past few years, the volume of the biomedical literature has been growing very fast, expanding by almost 1 million new scientific papers per year, indexed by MEDLINE. This fact makes the task of monitoring the knowledge and the changes in the biomedical domain extremely difficult. This in turn affects the maintenance of the existing biomedical ontologies, but in parallel motivates the creation of new, larger and more detailed thesauri in the domain, that may cover very different information needs.

Producing sufficient and concise answers from this wealth of information that exists in the biomedical domain is a challenging task for traditional search engines, which largely rely on term (keyword) indexing. Obtaining the required information is made even more difficult by non-standard terminology and the ambiguity of the technical terms involved. Therefore, indexing at the semantic (concept) level, rather than at the level of keywords only, is particularly important. Biomedical concept taxonomies or, more generally, ontologies are abundant and they provide concept inventories that can be used in semantic indices. Hierarchical classification algorithms [3] can classify documents and questions onto the concepts of these inventories, facilitating the matching of questions, documents, and also structured data (e.g., RDF triples) that already have explicit semantics based on the same concepts.

More specifically, for the two BIOASQ challenge tasks, the selection of resources follows the notion of the triangle *drug-target-disease* which defines the prime information axes for any medical investigation. This *“knowledge-triangle”* supports the conceptual linking of biomedical knowledge databases and the processing of the related resources. Based on this notion, systems can address questions that combine any path connecting the vertices of the triangle, provided that they can also annotate with accuracy the natural language questions with ontology concepts. From this perspective, Table 17 shows the candidate resources that were considered for inclusion in the BIOASQ challenge. The criteria based on which the selection was made are summarized into the following:

- avoid as much as possible the overlap between the selected resources,

**Table 17:** **The candidate resources that were examined for inclusion in the** BIOASQ **challenge by type**

**Focus**	**Resources**
Drugs	**Jochem**, Drug Ontology, ATC Ontology, DrugBank
Targets	**Gene Ontology**, **UniProt**, SuperTarget, Matador
Diseases	**Disease Ontology**, ICD-10, Diseasome
General Purpose	**MeSH**, SNOMED CT, UMLS
Document Sources	**PubMed**, **PubMed Central**
Linked Data	**LinkedLifeData**

Highlighted are the final selected resources.
- cover all aspects of the *drug-target-disease* associations,
- re-use as much as possible existing infrastructure from systems that have been already set up by the project consortium, e.g., GoPubMed, and,
- the included resources should be able to provide documents, snippets, concepts and triples (statements).

Based on the aforementioned criteria, highlighted in Table 17 are the selected resources: *Jochem* for drugs, GENE ONTOLOGY and UNIPROT for targets, DISEASE ONTOLOGY for diseases, MESH as a general purpose domain dictionary, PUBMED and PUBMED CENTRAL for documents, and LINKED LIFE DATA for triples (statements). A short description of the selected resources follows.

**Jochem** *Jochem* [53,54], the Joint Chemical Dictionary, is a dictionary for the identification of small molecules and drugs in text, combining information from MESH, CHEBI, DRUGBANK, KEGG, HMDB, and CHEMIDPLUS. The included resources were chosen on the basis of free availability. They are downloadable terminology databases containing small molecules from human studies. Given the variety and the population of the different resources merged in *Jochem*, it is currently one of the largest publicly available biomedical resources for drugs and chemicals.

**Gene ontology** The GENE ONTOLOGY [55] is currently the most successful case of ontology use in bioinformatics and provides a controlled vocabulary to describe functional aspects of gene products. The ontology covers three domains: *cellular component*, the parts of a cell or its extracellular environment; *molecular function*, the elemental activities of a gene product at the molecular level, such as binding or catalysis; and *biological process*, operations or sets of molecular events with a defined beginning and end, pertinent to the functioning of integrated living units: cells, tissues, organs, and organisms.

**UniProt** The *Universal Protein Resource* [56] (UNIPROT) provides the scientific community with a comprehensive, high-quality and freely accessible resource of protein sequence and functional information. Its protein knowledge base consists of two sections: *Swiss-Prot*, which is manually annotated and reviewed, and contains approximately 500 thousand sequences, and *TrEMBL*, which is automatically annotated and is not reviewed, and contains approximately 23 million sequences. The primary mission of UNIPROT is to support biological research by maintaining a stable, comprehensive, fully classified, richly and accurately annotated protein sequence knowledge base, with extensive cross-references and querying interfaces freely accessible to the scientific community. In particular the *Swiss-Prot* component of *UniProt* is a high-quality, manually annotated, non-redundant protein sequence database which combines information extracted from scientific literature and biocurator-evaluated computational analysis. The aim of *Swiss-Prot* is to provide all known relevant information about a particular protein. Annotation is regularly reviewed to keep up with current scientific findings. The manual annotation of an entry involves detailed analysis of the protein sequence and of the scientific literature.

**Disease ontology** The DISEASE ONTOLOGY [57] contains data associating genes with human diseases, using established disease codes and terminologies. Approximately 8,000 inherited, developmental and acquired human diseases are included in the resource. The DISEASE ONTOLOGY semantically integrates disease and medical vocabularies through extensive cross-mapping and integration of MESH, ICD, NCI’s thesaurus, SNOMED CT and OMIM disease-specific terms and identifiers. The DISEASE ONTOLOGY is utilized for disease annotation by major biomedical databases (e.g., ARRAY EXPRESS, NIF, IEDB), as a standard representation of human diseases in biomedical ontologies and as an ontological cross-mappings resource between MESH and OMIM. The DISEASE ONTOLOGY has been incorporated into open source tools (e.g., *Gene Answers*, *FunDO*) to connect gene and disease biomedical data through the lens of human diseases.

**The medical subject headings hierarchy** Medical Subject Headings [58] (MESH) is a hierarchy of terms maintained by the *United States National Library of Medicine* (NLM) and its purpose is to provide headings (terms) which can be used to index scientific publications in the life sciences, e.g., journal articles, books, and articles in conference proceedings. The indexed publications may be then searched through popular search engines, such as PUBMED or GOPUBMED, using the MESH headings to filter semantically the results. This retrieval methodology seems to be in some cases beneficial, especially when precision of the retrieved results is important [2]. MESH includes three types of data: (i) *descriptors*, also known as *subject headings*, (ii) *qualifiers*, and, (iii) *supplementary concept records*. *Descriptors* are the main terms that are used to index scientific publications. The *descriptors* are organized into 16 trees, and as of 2013 they are 26,853. They include a short description or definition of the term, and they frequently have synonyms, known as *entry terms*. *Qualifiers*, also known as *subheadings*, may be used additionally to narrow down the topic of each of the *descriptors*. In total there are approximately 80 *qualifiers* in MESH. *Supplementary concept records*, approximately 214,000 in the most recent MESH release, describe mainly chemical substances and are linked to respective *descriptors* in order to enlarge the thesaurus with information for specific substances. MESH is the main resource used by PUBMED to index the biomedical scientific bibliography in MEDLINE.

**PubMed and PubMed central** The primary corpora for text-based *QA* in the biomedical domain are accessible through PUBMED [59] and PUBMED CENTRAL [60]. PUBMED, a service provided by the *National Library of Medicine* (NLM), under the *U.S. National Institutes of Health* (NIH), contains over 23 million citations from MEDLINE, a bibliographic database (DB) of biomedical literature, and other biomedical and life science journals dating back to the 1950s. It is accessible through the *National Center for Biotechnology Information* (NCBI). PUBMED CENTRAL is a digital archive of full-text biomedical and life science articles. The full text of all *PubMed Central* articles is freely available, but not for bulk download. As of July 2011, the archive contains approximately 2.2 million items, including articles, editorials and letters.

**Linked life data** The *LLF* project [61] provides the *LinkedLifeData* platform. *LinkedLifeData* is a data warehouse that syndicates large volumes of heterogeneous biomedical knowledge in a common data model. The platform uses an extension of the RDF model that is able to track the provenance of each individual fact in the repository and thus update the information. It contains currently more than 8 billion statements, with almost 2 billion entities involved. The statements are extracted from 26 biomedical resources, such as PUBMED, UMLS, DRUGBANK, DISEASOME, and GENE ONTOLOGY. The statements are publicly available, and the project provides also a wide list of instance mappings.

## Additional files

Additional file 1
**An example of a participating system answering a question progressing through the** BIOASQ **tasks.** This pdf file presents an example of how one of the participating systems to the BioASQ competition is answering an input natural language question progressing through the BioASQ competition tasks. The output of the system for each of the tasks is compared with the “gold” answers, e.g., MeSH concepts, relevant documents, snippets, triples, and ideal answer to the input question.

Additional file 2
**Selected journal list for Task 1a test datasets creation.** This text file contains the list of the 1993 selected journals based on which the test datasets for Task 1a were created. Each line corresponds to a name of a journal indexed by PubMed. The scientific articles published in any of the journals in this list have an average annotation time with MeSH labels from the NLM curators of 90 days or less. The list can also be found online at: http://bioasq.lip6.fr/journals/.

Additional file 3
**Example of a** PUBMED **query that is used to generate a test dataset for** BIOASQ **Task 1a.** The text file contains an example of a PubMed query that is issued automatically by the respective BioASQ web service, in order to create a test data set for Task 1a. All three restrictions set for the generation of test datasets in Task 1a are included in this query. In this specific example, this query would generate a test dataset for the period 02/12/2013−16/12/2013.

Additional file 4
**Roll-out of the challenge.** The challenge resources are made available to the participants via the participants’ platform. The challenge data are being prepared by the experts via the tools and services of the BioASQ consortium based on guidelines, besides the data of Task 1a, which are based on the backlog of NLM’s documents which are still not annotated with MeSH concepts, and are retrieved automatically. The social network helps the experts to review the questions, and exchange comments. The challenge data are distributed to the participants in batches, with a limited time for submitting responses. The participants submit their answers via the participant’s platform, and results are produced automatically. In addition to the automated evaluation, experts review the answers of the systems based on several criteria, such as readability, and repetition.
