# Supplementary material for: An overview of the BIOASQ large-scale biomedical semantic indexing and question answering competition
Source: BMC Bioinformatics. 2015 Apr 30;16:138. doi: 10.1186/s12859-015-0564-6 (PMC4450488; doi:10.1186/s12859-015-0564-6)
Supplement: Additional file 1 — An example of a participating system answering a question progressing through the BIOASQ tasks. This pdf file presents an example of how one of the participating systems to the BioASQ competition is answering an input natural language question progressing through the BioASQ competition tasks. The output of the system for each of the tasks is compared with the “gold” answers, e.g., MeSH concepts, relevant documents, snippets, triples, and ideal answer to the input question. [file 12859_2015_564_MOESM1_ESM.pdf]

Resources (available to participants via BioASQ Tools and Services)

Databases and Ontologies: MeSH, GO, UniProt, Jochem, Disease Ontology (~ 880,000 concepts)

Linked Data: Linked Life Data Triples (~ 2,000,000,000 triples)

Documents: PubMed abstracts and subset of PMC full text articles (~ 22,000,000 PubMed entries and 800,000 full text articles)

Results of Task 1a

Evaluate the performance of WISHART on annotating PubMed abstracts with MeSH concepts.

18 Test datasets were distributed to the participants, in 3 batches of 6 datasets each.

Performance was measured on the basis of the Micro-averaged F Measure (flat measure), and the Leact Common Ancestor F Measure (hierarchical measure)

Results of WISHART S2 for the 3 batches are displayed in the table below:

Batch 1		Batch 2		Batch 3	
MiF	LCaF	MiF	LCaF	MiF	LCaF
0,463075	0,39735	0,506	0,4276	0,50675	0,42905

Access to resources via BioASQ services

Access to resources via the BioASQ Annotation Tool

BioASQ Participating System WISHART

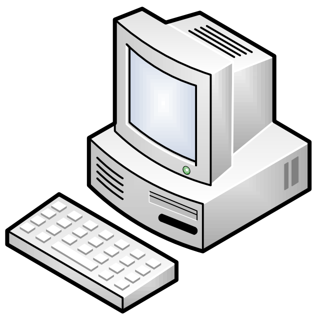

Annotation of documents with ontology concepts, and re-indexing of resources based on system needs

System receives question and type as input for Task 1b, Phase A

System receives question, type, golden documents, concepts, triples and snippets, as input for Task 1b, Phase B

System retrieves related documents, concepts, triples and snippets for input question

Preparation of benchmark questions by the BioASQ team of biomedical experts

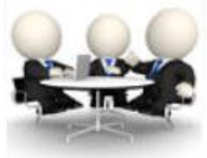

The BioASQ team of biomedical experts prepare the benchmark sets for Task 1b Phase A and Task 1b Phase B. For Phase A, they formulate the question, and synthesize the answer by retrieving related documents, concepts, and triples. In parallel, they annotate snippets of the related documents that are central to the question. Participating systems in Phase A receive as input the question and its type, and try to retrieve the 'gold standard' documents, concepts, triples and snippets. In Phase B they receive as input the 'gold standard' documents, concepts, snippets and triples, and provide as output the answer to the question,

Example Question: What is the biological role of expansins in fungi?
Type: Summary

System synthesizes the answer to the question by combining the information from the provided 'golden standard' documents, snippets, concepts and triples

Results of Task 1b Phase A

Evaluate the performance of WISHART on retrieving the 'golden' documents, concepts, triples and snippets related to the question.

System documents:

"http://www.ncbi.nlm.nih.gov/pubmed/23449921",
"http://www.ncbi.nlm.nih.gov/pubmed/12199698",
"http://www.ncbi.nlm.nih.gov/pubmed/23376652",
"http://www.ncbi.nlm.nih.gov/pubmed/23479888",
"http://www.ncbi.nlm.nih.gov/pubmed/23435902",
"http://www.ncbi.nlm.nih.gov/pubmed/23413712",
"http://www.ncbi.nlm.nih.gov/pubmed/23474124",
"http://www.ncbi.nlm.nih.gov/pubmed/15605243",
"http://www.ncbi.nlm.nih.gov/pubmed/19151131",
"http://www.ncbi.nlm.nih.gov/pubmed/18400936",
"http://www.ncbi.nlm.nih.gov/pubmed/23437139",
"http://www.ncbi.nlm.nih.gov/pubmed/19058186",
"http://www.ncbi.nlm.nih.gov/pubmed/23436216",
"http://www.ncbi.nlm.nih.gov/pubmed/23449077",
"http://www.ncbi.nlm.nih.gov/pubmed/23360472"

Golden documents:

"http://www.ncbi.nlm.nih.gov/pubmed/20478643",
"http://www.ncbi.nlm.nih.gov/pubmed/19479322",
"http://www.ncbi.nlm.nih.gov/pubmed/18406638",
"http://www.ncbi.nlm.nih.gov/pubmed/18400936",
"http://www.ncbi.nlm.nih.gov/pubmed/15605243",
"http://www.ncbi.nlm.nih.gov/pubmed/15195944"

System concepts:

"http://www.nlm.nih.gov/cgi/mesh/2012/MB_cgi?field=uid&exact=Find+Exact+Term&term=D005658",
"http://amigo.geneontology.org/cgi-bin/amigo/term_details?term=0009620",
"http://www.biosemantics.org/jochem#4249749",
"http://www.uniprot.org/uniprot/EXPB_PASNO",
"http://www.uniprot.org/uniprot/FIP1_CAEBR"

Golden concepts:

"http://www.nlm.nih.gov/cgi/mesh/2012/MB_cgi?field=uid&exact=Find+Exact+Term&term=D005658"

System snippets (2 examples/17 snippets submitted are shown):

{
"offsetInBeginSection": 1052,
"offsetInEndSection": 1194,
"text": "Cytoplasmic aggregation was only induced by contact with compatible fungi, whereas PPA appearance was specifically triggered by the AM fungus.",
"beginSection": "sections.0",
"document": "http://www.ncbi.nlm.nih.gov/pubmed/19151131",
"endSection": "sections.0"
},
{
"offsetInBeginSection": 1312,
"offsetInEndSection": 1473,
"text": "The up-regulation of an expansin-like gene, already identified as an early marker of AM fungal contact, was triggered in wild-type roots by all the fungi tested.",
"beginSection": "sections.0",
"document": "http://www.ncbi.nlm.nih.gov/pubmed/19151131",
"endSection": "sections.0"
},

System snippets (2 examples/7 golden snippets submitted are shown):

{
"beginSection": "sections.0",
"document": "http://www.ncbi.nlm.nih.gov/pubmed/19479322",
"endSection": "sections.0",
"offsetInBeginSection": 442,
"offsetInEndSection": 585,
"text": "To evaluate a putative implication of three newly identified expansin/family 45 endoglucanase-like (EEL) proteins in lignocellulose degradation"
},
{
"beginSection": "sections.0",
"document": "http://www.ncbi.nlm.nih.gov/pubmed/18406638",
"endSection": "sections.0",
"offsetInBeginSection": 1082,
"offsetInEndSection": 1232,
"text": "Our results show that EgID is a conidial cell wall localized expansin-like protein, which could be involved in cell wall remodeling during germination"
},

There were zero (0) triples returned by the system. In parallel, no relevant triples were retrieved and returned by the experts for this question.

Based on the above, the performance of the participating system in Task 1b Phase A for this specific question is shown in the following table.

Documents					Concepts					Snippets				
Precision	Recall	F Measure	MAP	GMAP	Precision	Recall	F Measure	MAP	GMAP	Precision	Recall	F Measure	MAP	GMAP
0.13	0.33	0.19	0.16	0.16	0,2	1.0	0.33	1.0	1.0	0.09	0.38	0.15	0.132	0.132

Results of Task 1b Phase B

Example Question: What is the biological role of expansins in fungi?
Type: Summary

System Answer: Alpha-Expansins are extracellular proteins that increase plant cell-wall extensibility. These wall-loosening proteins are directly involved in the accommodation of the fungus by infected cortical cells.

Golden Answer: Expansins are extracellular proteins that increase plant cell-wall extensibility. These wall-loosening proteins are involved in cell wall extension and polysaccharide degradation. In fungi expansins and expansin-like proteins have been found to localize in the conidial cell wall and are probably involved in cell wall remodeling during germination.

The system answer was scored by the experts as follows (all scores from 1 to 5, where 5 is the best score).

Recall: 4
Precision: 4
Repetition: 5
Readability: 5
