# Supplementary figures and images for: An overview of the BIOASQ large-scale biomedical semantic indexing and question answering competition
Source: BMC Bioinformatics. 2015 Apr 30;16:138. doi: 10.1186/s12859-015-0564-6 (PMC4450488; doi:10.1186/s12859-015-0564-6)

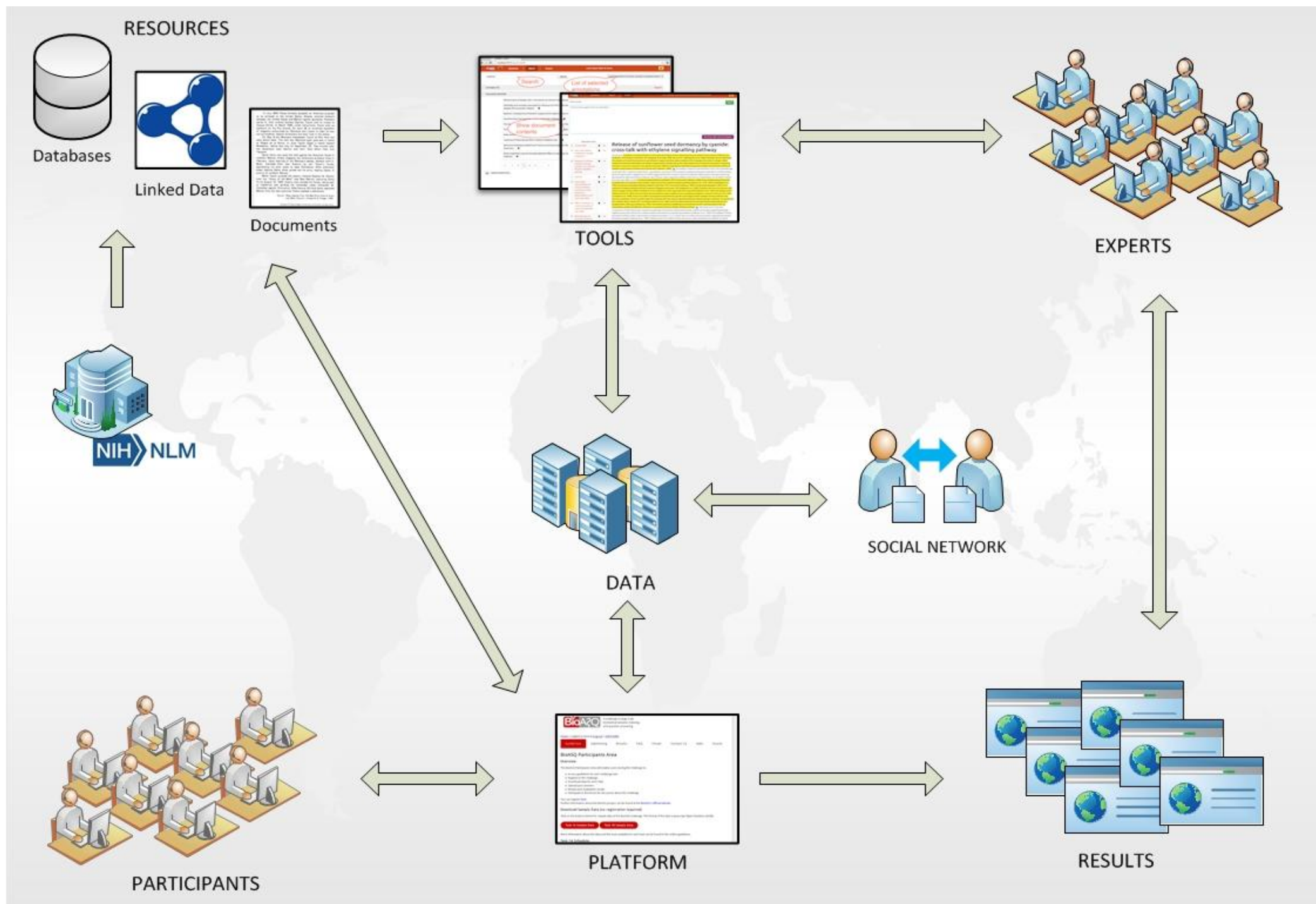

Supplement: Additional file 4 — Roll-out of the challenge. The challenge resources are made available to the participants via the participants’ platform. The challenge data are being prepared by the experts via the tools and services of the BioASQ consortium based on guidelines, besides the data of Task 1a, which are based on the backlog of NLM’s documents which are still not annotated with MeSH concepts, and are retrieved automatically. The social network helps the experts to review the questions, and exchange comments. The challenge data are distributed to the participants in batches, with a limited time for submitting responses. The participants submit their answers via the participant’s platform, and results are produced automatically. In addition to the automated evaluation, experts review the answers of the systems based on several criteria, such as readability, and repetition. [file 12859_2015_564_MOESM4_ESM.pdf]
